# Phyllosphere bacterial community dynamics in response to bacterial wildfire disease: succession and interaction patterns

**DOI:** 10.3389/fpls.2024.1331443

**Published:** 2024-03-12

**Authors:** Deyuan Peng, Zhenhua Wang, Jinyan Tian, Wei Wang, Shijie Guo, Xi Dai, Huaqun Yin, Liangzhi Li

**Affiliations:** ^1^ Zhangjiajie Tobacco Company of Hunan Province, Zhangjiajie, China; ^2^ School of Minerals Processing and Bioengineering, Central South University, Changsha, China; ^3^ Key Laboratory of Biometallurgy of Ministry of Education, Central South University, Changsha, China

**Keywords:** HGT (Horizontal gene transfer), phyllosphere, bacterial communities, wildfire disease, molecular ecological network

## Abstract

Plants interact with complex microbial communities in which microorganisms play different roles in plant development and health. While certain microorganisms may cause disease, others promote nutrient uptake and resistance to stresses through a variety of mechanisms. Developing plant protection measures requires a deeper comprehension of the factors that influence multitrophic interactions and the organization of phyllospheric communities. High-throughput sequencing was used in this work to investigate the effects of climate variables and bacterial wildfire disease on the bacterial community’s composition and assembly in the phyllosphere of tobacco (*Nicotiana tabacum* L.). The samples from June (M1), July (M2), August (M3), and September (M4) formed statistically separate clusters. The assembly of the whole bacterial population was mostly influenced by stochastic processes. PICRUSt2 predictions revealed genes enriched in the M3, a period when the plant wildfire disease index reached climax, were associated with the development of the wildfire disease (secretion of virulence factor), the enhanced metabolic capacity and environmental adaption. The M3 and M4 microbial communities have more intricate molecular ecological networks (MENs), bursting with interconnections within a densely networked bacterial population. The relative abundances of plant-beneficial and antagonistic microbes Clostridiales, Bacillales, Lactobacillales, and Sphingobacteriales, showed significant decrease in severally diseased sample (M3) compared to the pre-diseased samples (M1/M2). Following the results of MENs, we further test if the correlating bacterial pairs within the MEN have the possibility to share functional genes and we have unraveled 139 entries of such horizontal gene transfer (HGT) events, highlighting the significance of HGT in shaping the adaptive traits of plant-associated bacteria across the MENs, particularly in relation to host colonization and pathogenicity.

## Introduction

Plants harbor diverse microbial species playing crucial roles in their growth, health, and productivity (Bai et al., 2015; Xin et al., 2016; Brader et al., 2017; Ahmed et al., 2022). These microorganisms form co-evolved communities that contribute to disease protection and act as a supplement of the plant’s immune system. Apart from the root zone, recent researches have emphasized the influence of phyllosphere microbes on plant growth, including nitrogen fixation, plant pathogen control, and organic pollutant bioremediation (Vorholt, 2012; Xu et al., 2022).

The contact between a terrestrial plant’s aboveground section and the surrounding air is called the phyllosphere. It is estimated that the surface of approximately 100 million square kilometers of leaves harbors over 10^26^ bacteria globally, making the phyllosphere highly biodiverse habitats (Forero-Junco et al., 2022).

Traditionally, the phyllosphere has been considered an inhospitable habitat for microbial colonization due to prolonged exposure to solar/ultraviolet radiation, extreme diurnal temperature fluctuations, desiccation, humidity fluctuations, rain scouring, and limited nutrient availability (Truchado et al., 2017).

High-throughput sequencing methods have, however, recently made it possible to characterize the spatiotemporal organization of the phyllosphere microbiome in great detail. Numerous microorganisms with densities as high as 10^6^–10^7^ cells per square centimeter have been found to reside in the phyllosphere, according to these research (Kembel et al., 2014; Carvalho and Castillo, 2018; Ding et al., 2022). These microbes perform a variety of biological tasks, including as improving plant resistance to disease, biocontrolling phytopathogens, fixing nitrogen, breaking down toxic and hazardous materials, and producing plant hormones and volatile organic compounds (Vorholt, 2012; Xu et al., 2022). Furthermore, the phyllosphere’s suitability for experiments and visual examination makes it an appropriate model system for testing basic ecological ideas (Redford and Fierer, 2009; Remus-Emsermann and Schlechter, 2018).

Microorganisms, including phytopathogens, are found in intricate microbial communities within natural ecosystems (Brader et al., 2017; Hu et al., 2020; Ahmed et al., 2022). They interact with each other and with their host organisms or larger entities. However, comprehensive comprehension of multilateral interactions, particularly interactions within microbial communities, is in its infancy.

Plant disease development often involves collaborative efforts from various pathogens, commensal microorganisms, and abiotic factors. They directly affect host defenses and disrupt microbiota structures (Hajishengallis and Lamont, 2016). Auxiliary pathogens and commensal microbes, also identified as bacterial companions, facilitate the establishment of these pathogens in the community by exploiting compromised defenses, thereby enhancing their survival chances. Comprehensive understanding of these interactions is necessary for predicting disease incidence and severity and finding novel solutions against them. For example, Dai et al. (2022) investigate the spatiotemporal changes in community tobacco leaves infected by brown spot disease. The results of the investigation showed that *Pseudomonas, Sphingomonas*, and *Methylobacterium* all became more abundant proportional to the age of tobacco leaves. Similarly, Liu et al. (2022) showed that inoculating *Bacillus velezensis* SYL-3 suppressed diseases like *Alternaria alternata* and tobacco mosaic virus (TMV) while increasing beneficial bacteria like *Pseudomonas* and *Sphingomonas*.

Horizontal gene transfer (HGT) is of significant importance in the evolution and succession of microbial communities, particularly in the transmission and acquisition of genes among different organisms (Li L. et al., 2022). Through HGT, emerging pathogens can acquire new DNA fragments from other organisms, influencing their progression. This process is significant as it enables the rapid sharing of genes providing superior defense mechanisms among distantly related organisms, potentially facilitating processes like eco-invasion and adaptation to new environments. HGT occurs when a donor and recipient colonize a similar niche, promoting the colonization of novel microbial species in that niche. Consequently, HGT can lead to the emergence of microorganisms with altered pathogenicity and the development of entirely new pathogens (James et al., 2006; Richards et al., 2011; Chaib De Mares et al., 2015). Certain plant surface sites have been shown to be conducive to microbial growth and colonization, leading to localized increases in active cell densities. The aboveground plant compartments offer nutrient-rich environments that are particularly beneficial for HGT and have been characterized as “hot spots” for microbial HGT (Pinto-Carbó et al., 2016). HGT can also be augmented by different compound excreted from plants (Nielsen and van Elsas, 2001; Kay et al., 2002). The implications of HGT extend beyond individual organisms, shaping the dynamics and genetic makeup of microbial communities.

In this study, a field cultivating the model plant, cigar tobacco (*Nicotiana tabacum* L.) affected by wildfire disease caused by the commonly-seen phytopathogen *Pseudomonas syringae* (Xin et al., 2018) was used for investigation on the natural succession of phyllosphere bacterial communities. The objectives of this study were to (i) clarify taxonomic and functional changes in the phyllosphere bacterial communities under biotic (bacterial wildfire disease) and environmental stresses of four time periods, M1, M2, M3, and M4, which correspond to the months of June, July, August, and September in 2022; (ii) explore ecological networks of various time periods and deduce putative gene sharing events within communities to offer insights into the microbial interaction.

## Results

### Dissecting bacterial community in the cigar tobacco phyllosphere

**Figure 1:**
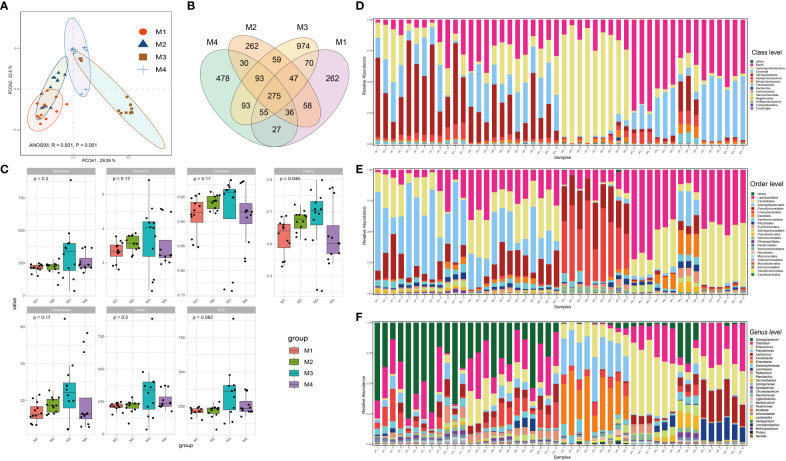
Bacterial community compositions and diversities of cigar tobacco phyllosphere. **(A)** Principal coordinate analysis (PCoA) illustrating the effects of different time series (T1, T2, T3 and T4) on the cigar tobacco phyllospheric bacterial community; **(B)** Venn diagrams illustrating number of shared or unique OTUs in each time series group (M1, M2, M3 and M4); **(C)** Alpha diversity indexes of cigar tobacco phyllosphere bacterial communities in each time series group (M1, M2, M3 and M4); **(D)** Bar chart illustrating bacterial community composition at the class level; **(E)** Bar chart illustrating bacterial community composition at the order level; **(F)** Bar chart illustrating bacterial community composition at the genus level.

The forty-eight phyllosphere bacterial DNA samples yielded 3,599 operational taxonomic units (OTU) and 2,347,083 high-quality paired 16S rRNA gene sequences (average: 48,896; range: 46,698–59,926 reads per sample). A respectable amount of reads for bacterial communities were acquired in all samples, according to rarefaction curves created to assess the richness of bacterial communities (**Supplementary Figure S1**). The microbiome from the four time series groups formed statistically separate clusters, according to principal coordinate analysis (PCoA) of Bray–Curtis distance. This suggests that the phyllospheric microbiome from different time periods displayed varied community compositions (**Figure 1A**). 51.9% of the total variation was explained by the first two axes combined (ANOISM, R = 0.601, P = 0.001). The four groups share 275 OTUs (the core taxa) in total; the M3 group contains the most unique OTUs (974), followed by the M4 group (478) (**Figure 1B**). Richness, Shannon, Simpson, Pielou, and invsimpson are examples of alpha diversity indices that clearly exhibited an upward trend from the M1 group to the M3 group before showing a modest decline at the M4 group (**Figure 1C**).

Alphaproteobacteria, Sphingobacteria, Clostridia, Gammaproteobacteria and Bacilli were the predominant classes (**Figure 1D**); Enterobacterales, Pseudomonadales, Sphingobacteriales, Clostridiales, Lactobacillales, Rhodospirillales, Alteromonadales, Flavobacteriales, Sphingomonadales, Burkholderiales, and Rhizobiales were the dominant bacterial orders (**Figure 1E**); Finally, *Pseudomonas, Stenotrophomonas, Enterobacter, Acinetobacter, Lactococcus, Enterococcus, Clostridium* and *Sphingobacterium* were the dominant bacterial genra (**Figure 1F**). Additionally, the bacterial community structure of cigar tobacco in current study is significantly distinct from that of fine-tuned tobacco that we previously reported (Wang Z. et al., 2022) upon partial least squares-discriminant analysis (PLS-DA), which aligns with the notion that host genotype affect the composition of microbial holobiont (**Supplementary Figure S2**). The PLS-DA model was validated by a permutation test: R2 intercept = 0.370 and Q2 intercept = -0.402. The PLS-DA models appeared to be reasonably predictable based on the negative Q2 intercept. Bacteria affiliated with *Sphingobacterium, Pantoea, Herminiimonas, Acinetobacter, Lactococcus, Enterococcus*, *Erwinia, Pseudomonas* and *Pediococcus* ranked the top 15 variable importance in projection (VIP), indicating them as the statistically significant taxa for the microbiota classification.

**Figure 2:**
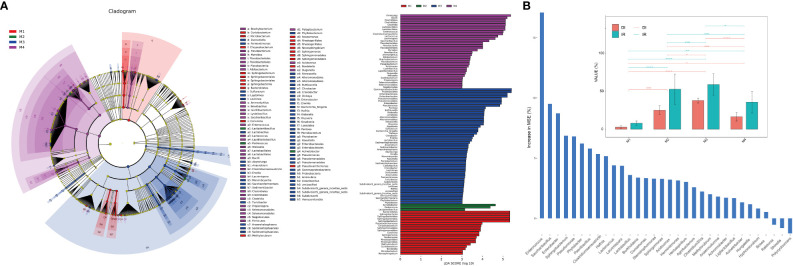
Analysis of microbial differences between groups. **(A)** The linear discriminant analysis effect size (LEfSe) analysis at species level of bacterial communities (with LDA score >3.1 and p < 0.05) among M1, M2, M3 and M4 groups presented by cladogram and distribution histogram; **(B)** Comparison of disease incidence rate (IR) and disease index (DI) among groups (top) with asterisks indicate significance; *p < 0.05; **p < 0.01; ****p < 0.001; ns, not significant. Random forest analysis showing genra contributed to the wildfire disease index (bottom).

**Figure 3:**
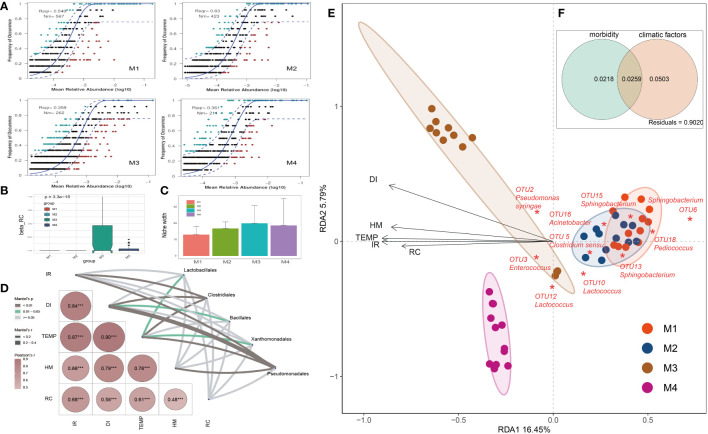
Analyses on factors that impacted the relative abundance and occurrence frequency of microbes in cigar tobacco phyllosphere. **(A)** Fit of the neutral community model (NCM) of community assembly. The OTUs more frequently present than predicted are in cyan, whereas those less frequently are in red. The blue dashed lines represent 95% confidence intervals around the model prediction and the OTUs fallen into the confidence intervals are regarded as neutrally distributed. Nm indicates the meta-community size times immigration, Rsqr indicates the fit to the neutral model. Neutral processes are the part within 95% confidence interval (red) while non-neutral are the parts including above and below prediction (dark green); **(B)** Comparison of incidence-based (Raup-Crick) beta-diversity (β_RC_) among groups; **(C)** Niche breadth comparison; **(D)** The partial Mantel tests showing the relationships between climate and disease indexes and plant associated microbes; Correlations were shown by the depth of colors, the significance showed with numbers; *p < 0.05; **p < 0.01; ***p < 0.001; **(E)** Redundancy analysis (RDA) of the relationships between bacterial community in tobacco leaves and environmental variables, including morbidity variables (disease incidence rate: IR, and disease index: DI) and climatic factors (temperature: TEMP, humidity: HM, and rainfall capacity: RC); **(F)** Variance partitioning analysis (VPA) showing contributions of morbidity and climatic variables to tobacco phyllospheric bacterial community variation.

LEfSe (Linear discriminant analysis Effect Size) analysis was used to identify the differential taxa in each group in order to further ascertain the changes in the makeup of the bacterial community throughout time series (**Figure 2A**). The importance of bacterial biomarkers in each group is positively connected with the linear discriminant analysis (LDA) score (**Figure 3**). In comparison, M1 group exhibits enrichment of genra such as *Sphingobacterium* (LDA =5.33), *Pseudoxanthomonas* (LDA =3.99), and *Sphingomonas* (LDA =3.83). *Sphingobacterium* and *Sphingomonas* are aerobic foliar and phytohormone-producing bacterium capable of protecting plants from foliar diseases caused by *Pseudomonas syringae* (the wildfire disease pathogen) via substrate competition (Vogel et al., 2012). *In vitro* plant growth-promoting characteristics, such as phosphate solubilization, IAA synthesis, and ACC deaminase activity, were demonstrated by *Sphingomonas* isolates (Jin et al., 2023).

At the same time, M2 group shows enrichment of bacterial genra such as *Acinetobacter* (LDA =4.98), *Pediococcus* (LDA =4.69), and *Lactiplantibacillus* (LDA =3.39). Notably, the M3 period, which comparatively has a higher disease incidence rate (IR) and disease index (DI) than other groups (**Figure 2B**), shows significant enrichment of many bacterial genra of opportunistic pathogens, including *Enterobacter* (LDA =5.21), *Pseudomonas* (LDA =5.16), *Pantoea* (LDA =4.43), *Klebsiella* (LDA =4.37), *Escherichia_Shigella* (LDA =4.08), *Dickeya* (LDA =4.07), *Erwinia* (LDA =3.79) and *Pectobacterium* (LDA =3.46). Consistently, the random forest analysis indicated that genra *Pseudomonas*, *Sphingobacterium*, *Enterobacter*, *Saccharibacillus* and *Enterococcus* contributed greatly (top 5 MSE score) to the wildfire disease index (**Figure 2B**). Lastly, M4 group shows enrichment of bacterial orders of Clostridiales (LDA =5.53), Lactobacillales (LDA =5.56), and Bacillales (LDA =4.32). These bacteria often considered to be plant-beneficial and antagonistic against phytopathogens (Ahmed et al., 2022; Jaffar et al., 2023).

An established technique for inferring stochastic processes associated with community assembly is the neutral community model (NCM), which has proven useful in the explanation of a number of ecological phenomena (Roguet et al., 2015). This model could quantify the significance of processes that are not easy to observe directly but might have a great impact on microbial communities (i.e., dispersal and ecological drift). In our study, the NCM has predicted 54.9%, 63.0%, 35.9% and 36.1% of the relation between the occurrence frequency of OTUs and the relative abundance for M1, M2, M3 and M4 groups, respectively (**Figure 3A**). Consistently, the incidence-based (Raup-Crick) beta-diversity (β_RC_) values increased rapidly at M3 and thereafter decreased at M4 period, but they remained within the ‘stochastic’ range (−0.95 < β_RC_ < + 0.95) (Vass et al., 2020) (**Figure 3B**). Consistently, the estimated niche width of M3 group is relatively greater than that of other groups (**Figure 3C**). Besides, the Nm value followed a gradual downtrend from M1 (567) to M4 (211), indicating that the species dispersal on tested plant phyllosphere decreased as time go by (**Figure 3A**).

The partial Mantel tests were used to explore the relationships between climate and disease indexes and plant associated microbes (**Figure 3D**). The compositions of main bacterial taxa such as those belonging to Pseudomonadales were significantly correlate with the disease index (DI, *r *> 0.4, *p* < 0.01). Redundancy analysis (RDA) was further applied to reveal the relationship between phyllospheric bacterial populations and factors (**Figure 3E**). RDA results showed that morbidity variables, including wildfire disease incidence rate (IR) and disease index (DI), are positively correlated with temperature (TEMP), humidity (HM) and rainfall capacity (RC). In addition, *Pseudomonas syringae* (OTU2), the pathogen of bacterial wildfire disease, and *Enterococcus* (OTU3), a kind of gram-positive and opportunistic pathogen (Dickel et al., 2018), were also positively correlated (contributing) to the disease index (DI). In the contrary, negatively correlated to the DI and these pathogenic taxa were genra such as *Acinetobacter, Sphingobacterium* and *Lactococcus*, indicating that they are the potential disease biocontrol agents (Ikeda et al., 2023; Jaffar et al., 2023). Overall, the morbidity (IR, DI) and climatic factors (TEMP, HM, RC) have significantly affected the phyllospheric bacterial community. Variance partitioning analysis (VPA) further showed that the complete set of the morbidity and climatic variables together could explain 2.5% of the variation of the tested phyllospheric bacterial communities, with climatic variables contributing more than morbidity (**Figure 3F**). The high proportion of unexplained variation in VPA also suggested the potential importance of neutral or stochastic processes during community assembly.

### The predicted function profiles of microbiomes are influenced by disease

**Figure 4:**
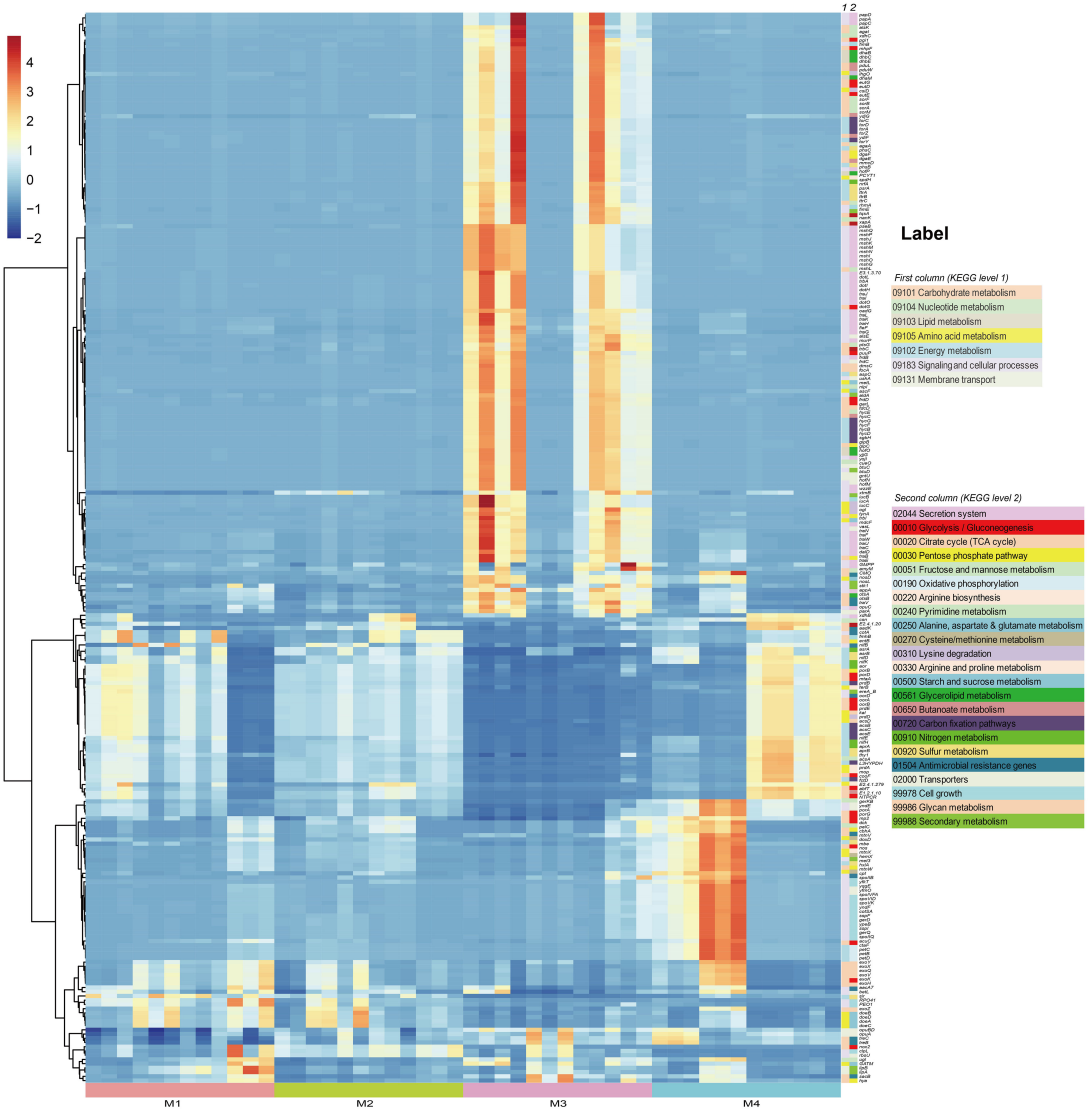
PICRUSt-predicted metagenome functions at KO level with significant different abundance among groups.

In order to investigate the effects of (a)biotic factors on the community functions of different periods, metagenomes of bacterial communities were predicted using PICRUSt2 and then annotated by referring to the KEGG database. A total of 7,281 KEGG Orthologs (KOs) were predicted in the phyllosphere-associated communities. PCoA analysis at the KO level showed that community functions of different time series significantly differed from each other (ANOSIM, R^2 = ^0.513, P < 0.001), suggesting that the bacterial wildfire disease also had a significant effect on microbiome functions of different time (**Supplementary Figure S3**). C, N, S cycling, secretion and adaption related genes showed a varied pattern among the phyllosphere bacterial communities (**Figure 4**).

Specifically, functional genes more abundant in the M3 microbiome were involved in Secretion system (acting as virulence factor, such as genes encoding the conjugal transfer pilus assembly proteins Tra and Hof), Carbohydrate metabolism (e.g. Pentose phosphate pathway, Amino sugar and nucleotide sugar metabolism) and Energy metabolism (e.g. Carbon fixation, Sulfur metabolism), as well as genes related with osmotic stress resistance (e.g. *otsAB, opuC*). We proposed that these genes enriched in the M3, a period when the plant wildfire disease index reached climax (**Figure 2B**), were related with the deterioration of wildfire disease (more secretion of virulence factor), the enhanced metabolic capacity and environmental adaption. Similar observation was also reported previously (Wang et al., 2022). In comparison, functional genes more abundant in the M4 microbiome were involved in Glycolysis/Gluconeogenesis (e.g. *porB* and *porD*), Glycan metabolism (e.g. *exoHKVQXY*) and Sporulation (e.g. *spoVK, yndF*, and *cotSA*). These sporulation genes may represent an adaptive strategy that enables bacteria to survive harsh environmental conditions (e.g., depleted nutrient on old leaves) for prolonged periods of time (**Figure 4**).

### Characteristics of microbial interaction through co-occurrence network

**Figure 5:**
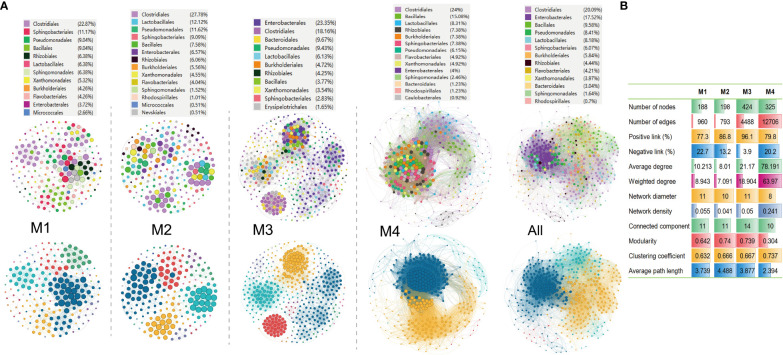
Molecular ecological networks of phyllospheric bacterial community of different groups. **(A)** Visualization of separate and overall molecular ecological networks. Each node represents an OUT; The size of each node is proportional to the number of connections (degree) and the colors of nodes represent different bacterial order (top) or module (bottom). The links between the nodes indicate strong and significant (*r >*0.7, *p* < 0.05) correlations; **(B)** Molecular ecological network properties of the four groups.

Molecular ecological networks (MENs) are built and visualized to investigate the impact of combinations of bacterial wildfire disease and climatic factors on microbial interactions across the distinct time periods. The aim was to gain a deeper insight into the interactions among phylospheric microorganisms. Analysis revealed that the four networks exhibited significantly different structures, highlighting the diverse impact of bacterial wildfire disease and climatic factors on microbial interactions across time (**Figure 5A**). This observation provides valuable insights into the dynamic nature of these interactions in response to environmental changes. The number of nodes in the MENs exhibits an increasing trend from M1 (188) to M3 (424), followed by a slight decrease at M4 (325). This tendency is also found in MEN properties such as the network diameter, modularity, average path length, and connected components (a maximal set of nodes such that each pair of nodes is connected by a path). In comparison, the edge numbers increase sharply from M1 (960) and M2 (793) to M3 (4,488) and M4 (12,706). Similar trend is also found in MEN properties such as average (weighted) degree, clustering coefficient, and network density (comparison between the edges available in a graph and a graph with all possible edges) (**Figure 5B**). The portion of positive correlation also followed an increase trend from M1 (77.3%) to M3 (96.1%). The positive correlation thereafter slightly decreased at M4 (79.8%).

Additionally, the average path lengths range from 2.39 to 4.49 (**Figure 5B**), exhibiting the network properties of typical small world with all nodes highly interlinked within the networks (Watts and Strogatz, 1998). The bacterial taxa Clostridiales (18.2%-27.8%), Pseudomonadales (6.2%-11.6%), Lactobacillales (6.1%-12.1%), Sphingobacteriales (2.8%-11.7%), Bacillales (3.8%-15.1%), Enterobacterales (3.7%-23.4%), Rhizobiales (4.3%-6.4%), Burkholderiales (4.3%-7.4%) always predominate the nodes of the MENs.

Following the results of MENs, we further test if the correlating bacterial pairs within the MEN have the possibility to share functional genes. Namely, the available genomes of phyllosphere bacterial isolates were used to detect putative HGT events from the correlating taxa in MENs. Interestingly, we have unraveled 139 entries of such HGT events (**Supplementary Table S1**) and we further perform phylogenetic reconstruction on representative horizontally transferred genes (HTGs) to verify the accuracy of HGT inferences.

**Figure 6:**
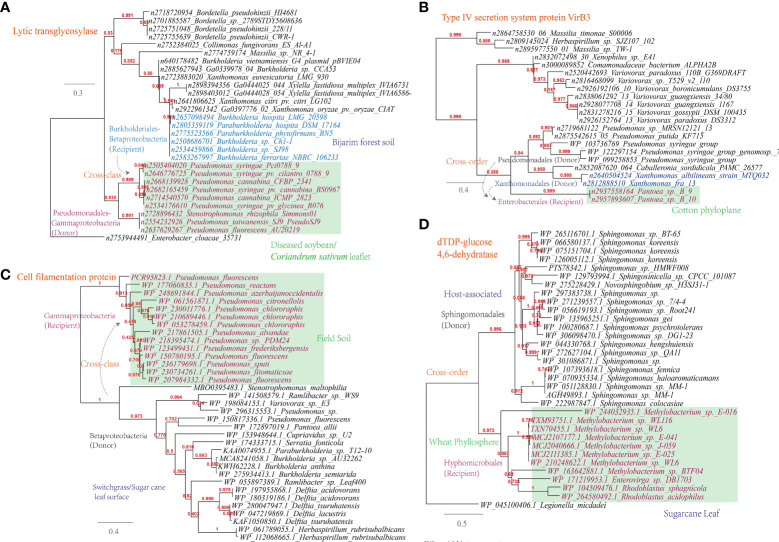
Phylogenetic analyses of the representative horizontally transferred genes related to host tissue entrance and nutrient utilization in this study (red color) with the closest sequences from Genbank database. The trees were constructed with PhyML; the branch length is proportional to the number of substitutions per site: **(A)** Lytic transglycosylase; **(B)** Type IV secretion system protein VirB3; **(C)** Cell filamentation protein; **(D)** dTDP-glucose 4,6-dehydratase.

These HTGs putatively confer adaptive functions to opportunistic plant-associated pathogenic microorganisms in the following categories:

(A) Enter/degrade host tissue:

Plant-associated pathogens degrade plant cell wall structures for energy and for gaining entry to the host. In current study, the identified HGT events related to these functions include:

(i) Lytic murein transglycosylase. This enzyme is found to be shared among plant-associated microbes of Pseudomonadales (e.g. the wildfire disease pathogen *Pseudomonas syringae*) from sampling sites like diseased soybean/*Coriandrum sativum* leaflet and Burkholderiales (e.g. the rhizosphere species *Burkholderia hospita*) from sampling sites like forest soil (**Figure 6A**). This is corresponding to 322 links in the overall MEN between *Burkholderia* and *Pseudomonas*. Lytic murein transglycosylase is a type of autolysin that exerts virulence by breaking the β-1,4 glycosidic bond between N-acetylmuramic acid and N-acetylglucosamine residues within the host peptidoglycan (Liu et al., 2012);

(ii) Type IV secretion system protein VirB3. This protein is found to be shared among plant-associated pathogens belonging to *Pseudomonas, Xanthomonas* and *Pantoea*, which inhabit the plant phyloplane and leaf surface, highlighting their potential role in intercellular communication and host manipulation (**Figure 6B**). This observation, corresponding to 541 links in the MEN among these three genra, underscores the significance of the type IV secretion system in facilitating pathogenic processes and contributing to disease (Christie et al., 2005). VirB3, which shares similarities with the pilin-like TraL protein involved in T-pilus assembly, represents a conserved component of this secretion system (Mossey et al., 2010).

In addition to the secretion system, other key mechanisms have been identified in plant-associated microbes. For instance, *Pseudomonas* spp. have been observed to utilize their flagellar apparatus to attach to and reach more favorable niches on the plant surface (Haefele and Lindow, 1987). This finding is consistent with the current study, which reveals the sharing of cell filamentation protein between Betaproteobacteria and Gammaproteobacteria strains (**Figure 6C**). Furthermore, the study identifies several other proteins, such as TrbB, TraO, TraD, and TraQ-like protein, which are involved in host attachment and virulence, and that are shared among plant-associated microbes in the MENs (**Supplementary Table S1**).

**Figure 7:**
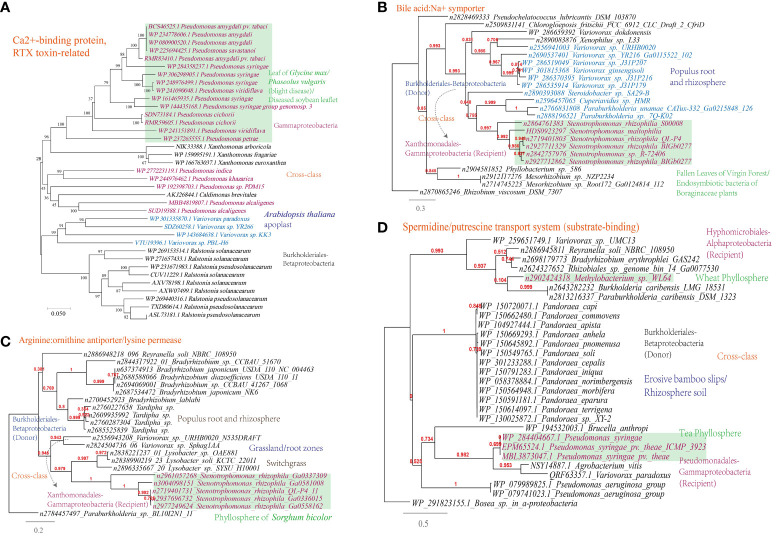
Phylogenetic analyses of the representative horizontally transferred genes related to responses to host defense in this study (red color) with the closest sequences from Genbank database. The trees were constructed with PhyML: **(A)** Ca^2+^-binding protein, RTX toxin-related; **(B)** Bile acid:Na^+^ symporter; **(C)** Arginine:ornithine antiporter/lysine permease; **(D)** Spermidine/putrescine transport system (substrate-binding).

Apart from the flagellar apparatus implicated in host attachment, it is also noteworthy that esterase/lipase genes, known for their involvement in cleaving the cuticle layer of the cell wall under the regulation of quorum-sensing, were found to be shared among plant-associated microorganisms like *Sphingomonas, Bradyrhizobium*, and *Paenibacillus* (**Supplementary Table S1**). Biofilm formation plays an important role in the colonization, abiotic resistance, and virulence of microbial community (Wang W. H. et al., 2022). Align with this, our study reveals the gene transmission of dTDP-glucose 4,6-dehydratase (RmlB) between plant-associated *Methylobacterium* and *Sphingomonas*, which are inhabitants of the wheat phyllosphere (**Figure 6D**).

(B) Ingest and utilization of nutrients from host:

A Ca^2+^-binding protein of the repeats in toxin (RTX) family, which causes the leakage of host cellular content through producing pore on the targeted cell membranes (Ostolaza et al., 2019), was found to be transferred among phytopathogens (**Figure 7A**).

**Figure 8:**
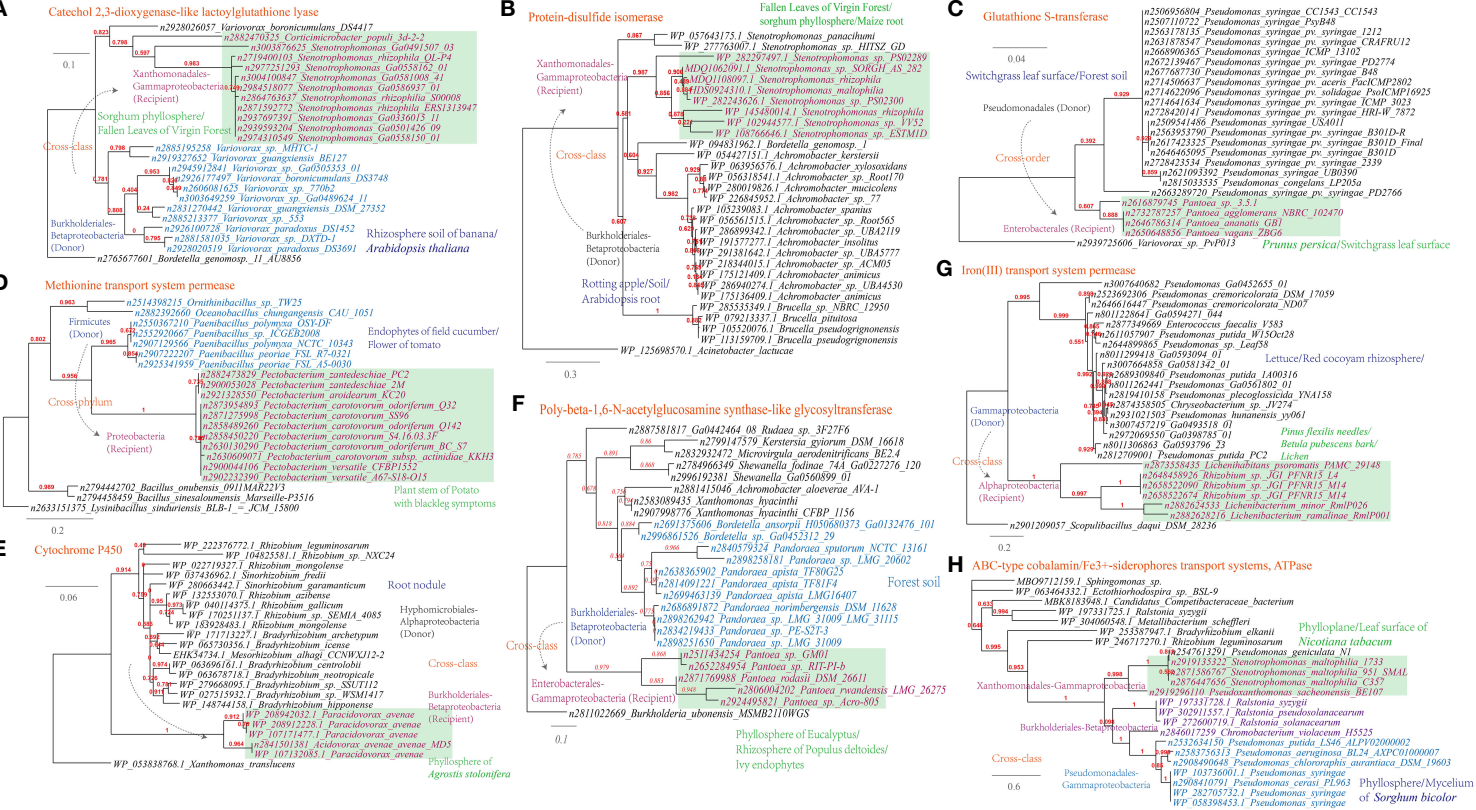
Phylogenetic analyses of the representative horizontally transferred genes related to responses to host defense in this study (red color) with the closest sequences from Genbank database. The trees were constructed with PhyML: **(A)** Catechol 2,3-dioxygenase-like lactoylglutathione lyase; **(B)** Protein-disulfide isomerase; **(C)** Glutathione S-transferase; **(D)** Methionine transport system permease; **(E)** Cytochrome P450; **(F)** Poly-beta-1,6-N-acetylglucosamine synthase-like glycosyltransferase; **(G)** Iron(III) transport system permease; **(H)** ABC-type cobalamin/Fe^3+^-siderophores transport systems, ATPase.

This study further supports the importance of HGT in shaping nutrient acquisition strategies in plant-associated microorganisms, as abundant HGT of transporter-encoding genes has been identified in microbes present in the MEN. The horizontally transferred genes identified are associated with the transport of nutrients including D-glucose, cellobiose, D-methionine, arabinose, nitrate/nitrite, raffinose/stachyose/melibiose, and more (**Supplementary Table S1**). To validate the representative horizontally transferred genes (HTGs), phylogeny inference was conducted on HTGs such as the bile acid:Na^+^ symporter (BASS) family (*Variovorax-Stenotrophomonas*) involved in the transport of Met-derived glucosinolates (Facchinelli and Weber, 2011), the spermidine/putrescine transport system (Hyphomicrobiales-Burkholderiales-Pseudomonadales) and the arginine:ornithine antiporter/lysine permease (Burkholderiales-Xanthomonadales) (**Figures 7B-D**).

(C) Counteract, Subvert, or Manipulate Host Pathways:

Upon recognizing an invading pathogen, plant cells activate multiple defense responses, including producing ROS (reactive oxygen species) and secreting antimicrobial toxins. Inhibition of the plant’s oxidative burst is crucial for the successful infection of several biotrophic and hemibiotrophic phytopathogens (Fu et al., 2022). In current study, HGT events of antioxidant enzymes that neutralize the reactive oxygen species were identified (**Supplementary Table S1**). For example, a catechol 2,3-dioxygenase was found to be shared between *Stenotrophomonas* and *Variovorax* that inhabit plant phyllosphere/fallen leaves (**Figure 8A**), in correspondence to 21 links in the MEN between these taxa; a protein-disulfide isomerase was found to be shared between *Stenotrophomonas* and *Achromobacter* that inhabit sorghum phyllosphere/maize root (**Figure 8B**), in correspondence to 30 links in the MEN between these taxa; a glutathione S-transferase was found to be shared between *Variovorax* and *Pseudomonas* that inhabit switchgrass leaf surface/forest soil (**Figure 8C**), in correspondence to 21 links in the MEN between these taxa. In addition, genes encoding peroxiredoxin (*Stenotrophomonas-Achromobacter*, 30 links in the MEN), ferredoxin (*Acidovorax-Bradyrhizobium*, a links in the MEN), Dyp-type peroxidase family (*Sphingomonas-Cupriavidus*, 52 links in the MEN), D-methionine (an antioxidant) transport system (**Figure 8D**, *Pectobacterium-Paenibacillus*, 338 links in the MEN) and non-heme chloroperoxidase (*Rhizobium-Sphingomonas*, 46 links in the MEN) were predicted to be shared by plant-associated microbes (**Supplementary Table S1**).

In order to overcome the inhibitory effects of antimicrobial toxins produced by plants, phytopathogens have developed strategies to break down these compounds using secreted enzymes (Maor and Shirasu, 2005). For example, the pea pathogen *Nectria haematococca* encodes a cytochrome P450 enzyme responsible for the detoxification of the pea-produced phytoalexin pisatin (Maloney and VanEtten, 1994), and its discontinuous distribution supports the hypothesis of HGT (Temporini and VanEtten, 2004). Consistently, genes encoding cytochrome P450 were identified to be shared between *Acidovorax* and *Rhizobium* from root nodule and the phyllosphere of *Agrostis stolonifera* (**Figure 8E**).

Glycosyltransferase is involved in the detoxification of organic toxics generated upon plant infections (Poppenberger et al., 2003; Sepúlveda-Jiménez et al., 2005). Consistently, genes encoding glycosyltransferase family were widely identified in plant-associated microbes (n=4, **Supplementary Table S1**). For example, phylogenetic analysis showed that species of Enterobacterales (Gammaproteobacteria) and Burkholderiales (Betaproteobacteria) that inhabit plant environments (phyllosphere/rhizosphere/endophyte) share a poly-beta-1,6-N-acetylglucosamine synthase-like glycosyltransferase via cross-class HGT (**Figure 8F**).

Siderophores are important in iron biogeochemical cycling in soils, pathogen competition, plant growth promotion and cross-kingdom signaling (Gu et al., 2020). Correspondently, components of cobalamin/Fe^3+^-siderophores transport systems and iron(III) transport system were found to be shared by pathogenic microbes inhabiting plant-associated environments, such as *Pseudomonas syringae* and *Ralstonia solanacearum* (**Figures 8G, H**). *Ralstonia solanacearum* is a soilborne phytopathogen that causes bacterial wilt and substantial yield losses in many plants (Yin et al., 2022).

(D) Abiotic Stresse Resistance

Plant-surface microorganisms frequently encounter challenging abiotic stresses due to fluctuations in climate, such as osmotic stress, sun exposure, and exposure to antimicrobial drugs like antibiotics and chemicals containing metal ions. HGT events play a crucial role in helping these microorganisms adapt to these harsh conditions (Li L. et al., 2022).

**Figure 9:**
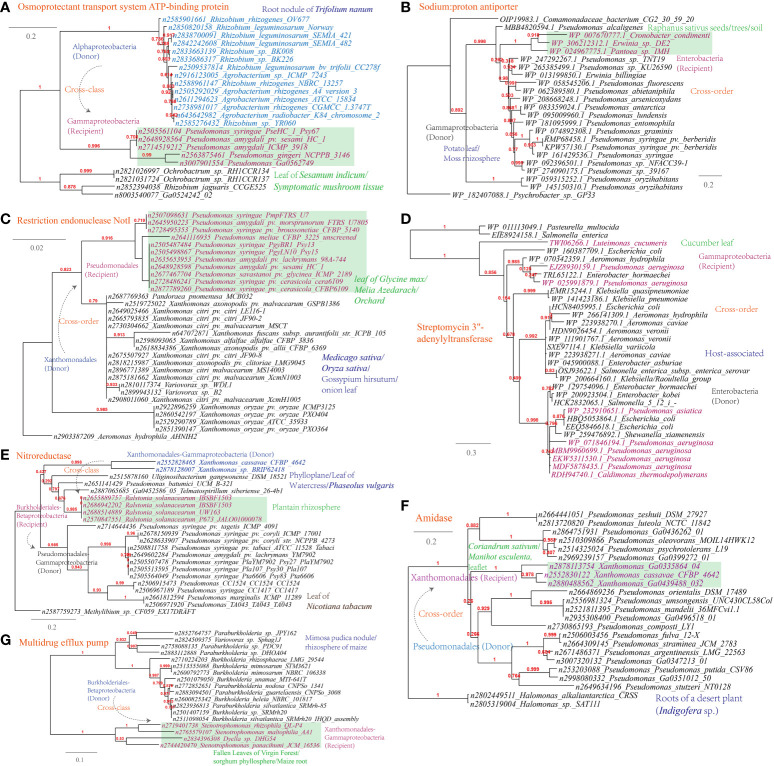
Phylogenetic analyses of the representative horizontally transferred genes related to responses to host defense in this study (red color) with the closest sequences from Genbank database. The trees were constructed with PhyML: **(A)** Osmoprotectant transport system ATP-binding protein; **(B)** Sodium:proton antiporter; **(C)** Restriction endonuclease NotI; **(D)** Streptomycin 3’-adenylyltransferase; **(E)** Nitroreductase; **(F)** Amidase; **(G)** Multidrug efflux pump.

Several examples of HGT events related to osmotic stress resistance have been identified (**Supplementary Table S1**). These HGT events include (**Figures 9A-C**): the osmoprotectant transport system ATP-binding protein (*Pseudomonas-Agrobacterium*, 338 links in the MEN), the sodium:proton antiporter (*Pseudomonas-Erwinia*, 445 links in the MEN), and the bile acid:Na^+^ symporter (*Variovorax-Stenotrophomonas*, 12 links in the MEN); HGT events associated with DNA repair and radiation resistance, such as the restriction endonuclease NotI (*Pseudomonas-Xanthomonas*, 319 links in the MEN); In terms of antimicrobial resistance, HGT events encompass a range of mechanisms involved in antibiotics inactivation and efflux (**Figure 9D-G**), such as streptomycin 3’-adenylyltransferase (*Luteimonas-Pseudomonas*, 319 links in the MEN), nitroreductase (*Xanthomonas-Pseudomonas*), a well-documented resistance factor (Müller et al., 2015), and amidase (*Xanthomonas-Pseudomonas*), as well as the multidrug efflux pumps (S*tenotrophomonas-Variovorax*).

**Figure 10:**
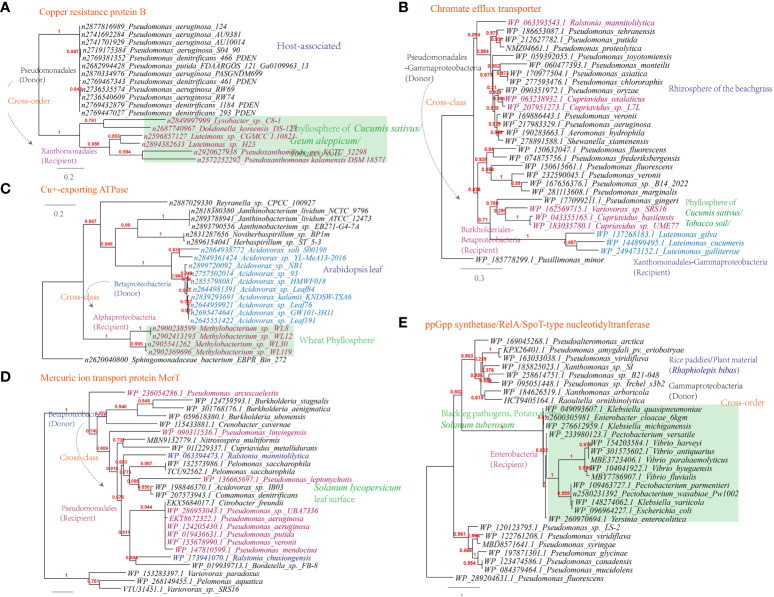
Phylogenetic analyses of the representative horizontally transferred genes related to abiotic stresse resistance in this study (red color) with the closest sequences from Genbank database. The trees were constructed with PhyML: **(A)** Chromate efflux transporter; **(B)** Copper resistance protein B; **(C)** Cu^+^-exporting ATPase; **(D)** Mercuric ion transport protein MerT; **(E)** ppGpp synthetase/RelA/SpoT-type nucleotidyltranferase.

Additionally, genes conferring resistance to antimicrobial chemicals containing metal ions, such as the chromate efflux transporter (*Luteimonas-Pseudomonas*), copper resistance protein B (*Luteimonas-Pseudomonas*), Cu^+^-exporting ATPase (*Methylobacterium-Roseomonas*, 6 links in the MEN) and mercuric ion transport protein MerT (*Ralstonia-Pseudomonas*, 324 links in the MEN), have also been horizontally transferred (**Figure 10A-D**).

Furthermore, the ppGpp synthetase/RelA/SpoT-type nucleotidyltranferase that participates in the stringent response is shared between *Pectobacterium* and *Pseudomonas* (326 links in the MEN); the starvation-inducible DNA-binding protein is shared between *Rhizobium* and *Sphingomonas*; The transcriptional regulator (MerR) is shared between *Stenotrophomonas* and *Sphingobium* (22 links in the MEN), as well as between *Xanthomonas* and *Pseudomonas* (319 links in the MEN). These proteins are additional examples of HGT events related to holistic response to abiotic stress resistance (**Figure 10E**).

## Discussion

*Pseudomonas* and *Enterobacter* affiliated microbes could be a primary factor that affecting the plant disease development. It was demonstrated that the two highly conserved effectors of *Pseudomonas syringae*, AvrE and HopM1, require high moisture on establishing the aqueous apoplast (Xin et al., 2016). In the phyllosphere, members of the *Enterobacter* genus can reproduce quickly, form aggregates when there is plenty of moisture, and respond sensitively to changes in the humidity on plant surfaces (Brandl and Mandrell, 2002; Brandl, 2006; Whipps et al., 2008). The Enterobacterial genra, *Pectobacterium* and *Dickeya*, are well-characterized plant pathogens that cause blackleg in tobacco and soft rot in Chinese cabbage, potato, and other crops (Ma et al., 2007). Consistently, these taxa were significantly enriched in the M3 period. On the other hand, Bacterial populations associated with the Clostridiales, Lactobacillales, and Bacillales orders have been identified as beneficial for plant growth. The recruitment of Bacillales in plant associated niche was reported to be induced by the plant exudation for disease suppression (Zhou et al., 2023). The existence of these bacteria in the M4 period may represent tobacco’s response mechanism to attract microorganisms during biotic stressors like pathogen invasion and unfavorable climatic conditions (Chen et al., 2014). Similarly, the relative abundances of Clostridiales, Bacillales, Lactobacillales (Ahmed et al., 2022; Jaffar et al., 2023), and Sphingobacteriales (Ikeda et al., 2023), which are often considered to be plant-beneficial and antagonistic microbes, showed significant decrease in severally diseased sample (M3) compared to the pre-diseased samples (M1/M2) in the MENs. This might be seen as the plant’s “cry for help” mechanism to lessen the effects of these stresses (Wang and Song, 2022).

The NCM has successfully predicted a significant portion of the community variance, indicating that stochasticity plays a more crucial role than determinism in shaping the tobacco phyllospheric bacterial community. Specifically, M1 and M2 groups demonstrated a higher NCM prediction fraction compared to M3 and M4 groups, suggesting that stochasticity is dominant in the early stages of community establishment, while deterministic processes become increasingly important over time in plant-related habitats (Dini-Andreote et al., 2015). This can be attributed to the critical influence of amino acids on stochastic processes in plant leaves for environmental selection (Xu et al., 2023). MENs of M3 and M4 demonstrate increased complexity, with abundant interactions in a highly connected microbial community. This suggests an increase in cooperative and facilitative interactions between pathogens and compatible microbes, potentially contributing to disease development (Ahmed et al., 2022). This could be attributed to the peak in rainfall and humidity during the M3 period (corresponding to August) at the sampling site, as indicated by data from the national meteorological center of China (http://www.nmc.cn/). These conditions likely provided an optimal environment for microbial growth and the formation of connections. We further analyze the structural robustness of MENs (M1, M2, M3, and M4) by calculating their natural connectivity (**Supplementary Figure S4**). A higher natural connectivity indicates a more structurally robust network (Li F. et al., 2022). Considering that real networks may experience node failures, we simulate random node failures by randomly removing nodes in our analysis. The natural connectivity of M1, M2, M3, and M4 in a network without node failures is 0.24, 0.27, 0.66, and 0.21, respectively. When approximately half of the nodes are removed, the natural connectivity of M3 is 61.5%, 68.0%, and 61.7% higher than that of M1, M2, and M4, respectively. This indicates that M3 topology possesses superior structural robustness and is more stable compared to other topologies. The natural connectivity algorithm reflects the communication links between nodes in reality. As a result, M3 topology’s higher structural robustness is expected to translate into better performance in real-world networks, where nodes may fail due to various reasons. In contrast, M1, M2, and M4 topologies lack the redundancy of M3. When nodes fail in these topologies, there are fewer alternative paths for nodes to take, leading to a more significant disruption of communication. Overall, the superior structural robustness of M3 makes it a more reliable and stable topology for various network applications. Consistent with our findings, a study by Hu et al. (2020) revealed that the rhizosphere and endophytic compartments of infected tobacco were more complex compared to those of healthy tobacco. Similarly, a higher level of connections was also observed in the wilt-diseased rhizoplane of tobacco compared to healthy samples (Ahmed et al., 2022; Tao et al., 2022).

HGT may also be significant in shaping the genetic repertoire and adaptive traits of plant-associated bacteria across the MENs, particularly in relation to host colonization, and pathogenicity. We have unraveled 139 entries of HGT events between the correlating bacterial pairs within the MEN that suggest the possibility to share functional genes. The validity of the HGT direction and identification during the inference process may be concerns. To allay these worries, we have included in our analysis the “gold standard” confirmation for the identified HGT genes: phylogenetic incongruence, which occurs when the evolutionary tree of a given protein family differs from the known organismal phylogeny, indicates that a given gene has been acquired through HGT from a different lineage (Schonknecht et al., 2014). For instance, the well-supported phylogenetic tree of methionine transport system permease illustrates that the protein sequences from *Pectobacterium* sp. (Proteobacteria) embedded within sequences from *Paenibacillus* sp. and *Bacillus* sp. (Firmicutes) (**Figure 8D**). Cross-phylum HGT originating from Firmicutes to Proteobacteria is the most economical explanation for this phylogenetic pattern.

Our study has identified abundant virulence-related HTGs. Consistently, there is evidence supports the notion that enzymes that degrades cell wall are often horizontally transferred among microbes to interact with host plants, as shown in previous studies (James et al., 2006; Richards et al., 2011; Chaib De Mares et al., 2015). The plant wildfire pathogen *Pseudomonas syringae* has been observed to involve prophages in transferring genes that encode Type III secreted effector (T3SE) proteins, which contribute to the evolution of pathogenic virulence (Hulin et al., 2023). Furthermore, the existence of Type IV secretion systems in transmissible bacterial plasmid with diverse excretion functions, including the delivery of toxic proteins by pathogens, further emphasizes their versatility and significance in host-pathogen interactions (Tauch et al., 2002). The presence of exogenously acquired DNA and pathogenicity determinants in the genome of the plant wilt pathogen *Ralstonia solanacearum* further emphasizes the role of HGT in the evolution of plant-associated pathogens (Salanoubat et al., 2002). Likewise, genomic differences correlated with virulence and host-range functions have been identified in phytopathogenic *Xanthomonas* species, underscoring the importance of HGT in shaping niche-adaptive traits (Da Silva et al., 2002). In the case of *Pectobacterium atrosepticum*, planta colonization promotes the transfer of integrative and conjugative elements, and leads to the acquisition of virulence genes (Vanga et al., 2015). Moreover, HGT has been proposed as a contributing factor to the adaption of *Erwinia tracheiphila*, the bacterial wilt pathogen affecting cucurbits (Shapiro et al., 2016). The prediction of the transmission of an alpha-1,2-mannosidase between *Stenotrophomonas* and *Sphingomonas* (75 links in the MEN, **Supplementary Table S1**) and cell-wall-degrading enzymes in current study is also noteworthy. This mannitol metabolic enzyme associates with growth and pathogenicity in phytopathogens (Vélëz et al., 2008) and can trim host glycoproteins (Reichenbach et al., 2018). Its exclusive transfer among distantly related phytopathogenic fungi further supports the significance of this enzyme (Qiu et al., 2016). The findings align with previous researches that highlight the function of these genes in the enzymatic modification and breakdown of the cell wall and to evade plant defenses (Voigt et al., 2005; Devescovi et al., 2007; Feng et al., 2009; Liu et al., 2018). RmlB is an enzyme associated with the formation of rhamnose-containing biofilms and the virulence of pathogenic bacteria (Sen et al., 2011). This prediction corresponds to the presence of 50 links in the MEN. Plant-associated microbes generally detect and respond to signals received from plants, including organic acid and sugar from exudate, and begin to colonize. Microorganisms utilize their flagella to navigate towards the plant once a signal is detected. Subsequently, bacteria adhere to the plant surface and form the biofilms (Delmotte et al., 2009). These findings underscore the multifaceted strategies employed by plant-related microorganisms to interact with their host plants. Biofilm formation facilitates the establishment and persistence of microbial communities, contributing to colonization, abiotic resistance, and virulence. Meanwhile, chemotaxis allows microorganisms to detect and respond to plant signals, aiding in their movement towards the plant surface. The presence of genes associated with flagella assembly, bacterial motility, and biofilm formation further supports the concept of biofilm-mediated colonization and the formation of complex microbial communities in the phyllosphere and stem settings. These collective adaptations enable plant-associated microbes to thrive and interact synergistically with their plant hosts.

The availability of nutrient compounds on leaves plays a crucial role in the colonization of the phyllosphere by bacterial populations (Lindow and Brandl, 2003). The release of small quantities of nutrients, including simple sugars like glucose, fructose, and sucrose, from the plant’s core has been observed (Lindow and Brandl, 2003). Consistently, we have detected abundant nutrient transport related HTGs. the nutrient acquisition strategies of plant-associated osmotrophic microbes heavily rely on the array of plasma membrane transporters they encode. Through HGT, these microbes can acquire novel transporter genes, expanding their nutrient repertoire and enabling them to occupy new ecological niches, ultimately giving them competitive advantages over other microorganisms from the same niches (Richards and Talbot, 2013). Polyamines like arginine and putrescine, in addition to serving as a source of essential and osmoprotectant amino acids, also function as signaling molecules that enable the microbiome to detect the presence of eukaryotic hosts (Jiménez-Bremont et al., 2014; Liu et al., 2018). Plant-associated microbial species alter their lifestyle upon detecting these compounds, promoting adhesion and biofilm development as a means to evade plant defenses. This response allows the microbes to establish a closer relationship with their host plants by forming biofilms that enhance their persistence and resistance to plant immune mechanisms (Jiménez-Bremont et al., 2014; Liu et al., 2018). This highlights the importance of HGT in shaping the evolution of microbial nutrient acquisition strategies, and suggests that plant-associated microbes may share common mechanisms for acquiring nutrients from their environments (Bachhawat et al., 2013).

Notably, the identified HGT events frequently occur within plant-associated environments, such as the phyllosphere and root. For instance, the sequences of *Stenotrophomonas* spp. inhabiting sorghum phyllosphere and maize root are clustered with *Burkholderia*-affiliated species from similar habitats as seen in the phylogeny of multidrug efflux pump (refer to **Figure 9G**). This can be explained by the consistent inheritance of conserved core plant microbiomes (holobionts) through seed dispersion or recruitment from the surrounding environments (Agler et al., 2016). Root microbiomes can rapidly spread to the endorhizosphere through fissures in lateral-root connections or wounds caused by phytopathogen (de Santi Ferrara et al., 2012). Endophytic bacteria may migrate from root to phyllosphere, where they can develop into local communities (Chi et al., 2005). In fact, extensive taxonomic and functional overlaps between the plant leaf and root microbial communities were unraveled (Bai et al., 2015). SourceTracker analysis of tea microbiome revealed high similarities (34%) within the phyllosphere and within the rhizosphere, indicating exchanges between roots and leaves (Xu et al., 2023). These microbial migration and colonization processes provide opportunities for HGT among plant holobionts, as demonstrated by the phylogenetic proximity of the HGT-affected genes. In addition, HGT can be stimulated by various exuded compounds, such as organic acids and amino acids (Nielsen and van Elsas, 2001; Kay et al., 2002).

Overall, these findings emphasized the shared features and mechanisms utilized by plant-associated microorganisms to interact with their host plants. The presence of shared proteins involved in cellular attachment, virulence, and enzymatic breakdown of the cell wall suggests the conserved strategies and adaptations in the microbial ecology of plant-associated pathogens.

## Materials and methods

### Disease incidence of bacterial wildfire disease

The incidence of bacterial wildfire disease in tobacco was assessed using the standards outlined in the tobacco pest classification and survey methods (GB/T 23222–2008) of China. The disease incidence was determined by calculating the percentage of diseased tobacco in each field. Additionally, the disease index was calculated using the formula:


Disease index (DI) = [Σ (r×N)/(n×R)]×100

where r represents the disease severity, N is the number of infected tobaccos with a rating of r, n is the total number of tobaccos tested, and R is the highest disease severity value in each field. Meteorological data were obtained from the National Meteorological Center of China’s website (http://www.nmc.cn/).

### Leaf collection method

Leaf samples of cigar tobacco (*Nicotiana tabacum* L) were collected from the Yongding Region, Sangzhi County, Zhangjiajie City, Hunan Province, China (29°28′3″N, 110°57′51″E) at four different time points in 2022: June (M1), July (M2), August (M3), and September (M4). Samples were taken from cigar tobacco fields with varying levels of bacterial wildfire disease, with three plants displaying typical symptoms of the disease sampled from each plot, resulting in a total of three duplicates. The sampling followed a random block pattern, covering a plot area of 90 m^2^, adhering to local planting methods. A total of 18 plants per plot were sampled. To extract foliar microbial DNA, middle leaves from every sixth plant were collected and stored at 4°C until transportation back to the laboratory.

### DNA extraction and high-throughput sequencing

To extract the genomic DNA of foliar microorganisms, we collected 15 grams of leaf samples from different areas of the leaf surface, specifically excluding the main and branch veins, using a sterile puncher. The samples were then placed in a 50 mL solution of 0.1% Tween-80 bacterial phosphate buffer (pH 7.0) and shaken for 30 minutes at 170 revolutions per minute (rpm) and 28°C. However, the current processing method for our samples may not accurately differentiate between endophytes and epiphytes. The resulting bacterial suspension was collected, and the leaf samples were washed twice more. Afterward, the collected suspensions were centrifuged for 15 minutes at 4°C and 10,000 rpm to pellet the microorganisms, which were then washed three times and re-suspended in sterile water. Subsequently, the microorganisms were resuspended in 1 mL of sterile water for DNA extraction, which was performed using the Bacteria Genomic DNA Kit according to the manufacturer’s protocol. The 16S rRNA gene’s V3/V4 regions were amplified using the specific primer pair 341F (5’-CCT ACG GGN GGC WGC AG-3’) and 805R (5’-GAC TAC HVG GGTATC TAA TCC-3’). Finally, the amplicons were sequenced using the Illumina NovaSeq PE250 platform by LC-Bio Technology Co., Ltd (Hang Zhou, Zhejiang Province, China).

### Sequencing processing and statistical analyses

We performed several steps to process the raw sequences. Firstly, the raw sequences were divided into sample libraries using barcodes. Subsequently, low-quality sequences with a quality score (QC) below 20 over a 5-base pair window size were removed using Btrim (Kong, 2011), and sequences shorter than 100 base pairs were eliminated. Then, the forward and reverse sequences were merged. Any sequences containing ambiguous bases or of incorrect length were excluded, and the remaining sequences were compared against the UNITE v8.2 (Kõljalg et al., 2005) to identify and remove possible chimeras.

Following this, the sequencing fragment lengths were restricted to 200–400 base pairs. The UPARSE (Edgar, 2013) was utilized to cluster and generate operational taxonomic units (OTUs) at a 97% similarity level. To ensure data authenticity, OTUs represented by only one sequence across the entire dataset (global singletons) were removed. All statistical analyses and calculations were conducted using the R (v 3.6.3) statistical platform (www.r-project.org).

Subsequently, we used analysis of similarities (ANOSIM) to assess significant differences in community dissimilarity and conducted a classification random forest analysis (Yuan et al., 2021) using the R randomForest package to identify key taxa. We also utilized the incidence-based (Raup-Crick) beta-diversity (β_RC_) to differentiate between deterministic and stochastic assembly processes (Chase, 2010; Stegen et al., 2013; Vass et al., 2020). In addition, we adopted a neutral community model (NCM) to predict the relationship between OTU detection frequencies and their relative abundance across the wider metacommunity, using R (version 3.6.3) to determine the potential importance of stochastic processes on community assembly.

### Network construction and HGT identification

To construct molecular ecological networks (MENs), we calculated correlations between pairwise OTUs that were present in over half of the samples using the SparCC method (Friedman and Alm, 2012). Only edges with a significant correlation higher than 0.7 (p < 0.01) were retained for network construction. We then evaluated the robustness of the microbial association networks against random and targeted node removals (Albert et al., 2000), using natural global network connectivity as a reliable measure of network robustness (Wu Jun et al., 2010). By sequentially removing nodes from the network, we observed how the natural connectivity of the microbial network changed, providing insights into its robustness.

Subsequently, following the results of MENs, we investigated if correlating bacterial pairs within the MENs had the potential to share functional genes. To achieve this, we utilized available genomes of phyllospheric bacterial isolates to detect putative horizontal gene transfer (HGT) events from the correlating taxa in MENs. Identification of horizontally transferred genes in the genomes of bacterial isolates from the phyllosphere was conducted using the Integrated Microbial Genomes Annotation Pipeline (IMGAP) v.5.0 (Markowitz et al., 2010). Genes in tested genomes were defined as having been horizontally transferred from a distant lineage based on the principle that genes had the best BLASTP hits (highest bit scores) or >90% of the best hits found outside the taxonomic lineage of the tested genome (i.e., from a distant phylum, class, etc.) and with lower-scoring hits or no hits within the lineage.

Furthermore, the phylogeny of various abiotic resistance genes was constructed based on gene-translating protein sequences using the PhyML v.3.0 program (Guindon et al., 2010) with the Maximum Likelihood (ML) method and 1,000 bootstrap replicates. These sequences were aligned with MUSCLE (Edgar, 2004) and trimmed with Gblocks (Talavera and Castresana, 2007) before tree construction, which was then visualized using iTOL (Letunic and Bork, 2021).

## Conclusions

Pathogenicity is a multifaceted concept influenced by various factors, including pathogen and host genotypes, environmental stresses, and microbial interactions. Together, these factors determine the plant’s response to disease-causing microorganisms. Traditional plant pathology textbooks typically describe the development of plant diseases as reliant on prerequisites such as a microbial pathogen with virulence or pathogenicity factors, a susceptible plant host, and environmental conditions that favor disease progression, such as humidity and temperature. However, recent recognition of the symbiotic or mutualistic relationships between many plants and microorganisms has led to a more nuanced understanding of disease causation. The hypothesis of a pathological microbiome suggests that pathogens are integrated into their biological environment, and the microbiome plays a crucial role in plant health and resistance to pathogens.

Taking into account the complexity of these interactions, a conceptual framework known as the disease tetrahedron has been proposed to encompass the interactions between disease determinants, using biological factors as a fourth dimension. In this framework, disease progression is influenced and moderated by various factors, including vectors that transmit pathogens, environmental factors, and the microbiota that regulate pathogenesis and plant defense. Environmental factors affect not only the host plant but also the pathogens and other biological factors, while biological factors directly influence plant performance, pathogens, and other biological components.

The study’s findings revealed distinct clusters formed by four series groups (M1, M2, M3, and M4) within the bacterial community, with the assembly primarily driven by stochastic processes. Predictions using PICRUSt2 showed that genes enriched in the M3 group were associated with disease progression, increased virulence factor secretion, enhanced metabolic capacity, and environmental adaptation. The molecular ecological networks of M3 and M4 demonstrated a higher complexity with numerous interactions within a highly connected microbial community.

The study also found that the abundance of beneficial plant microbes and antagonistic microbes decreased significantly in severely diseased samples (M3) compared to pre-diseased samples (M1/M2), indicating potential implications for disease progression. Furthermore, the study explored the potential for HGT within the bacterial pairs in the MENs and identified 139 instances of such HGT events.

Overall, the study provides valuable insights into the bacterial communities in the phyllosphere, shedding light on the dynamics of plant-microbe interactions. These findings contribute to the development of strategies to manage diseases, promote plant health, and engineer microbiomes to enhance the resilience of plants against foliar bacterial diseases.

## Data availability statement

The datasets presented in this study can be found in online repositories. The names of the repository/repositories and accession number(s) can be found below: BioProject, PRJNA1033823

## Author contributions

DP: Supervision, Writing – review & editing. ZW: Funding acquisition, Writing – review & editing. JT: Writing – review & editing. WW: Writing – review & editing. SG: Writing – review & editing. XD: Writing – review & editing. HY: Writing – review & editing. LL: Conceptualization, Methodology, Writing – original draft, Writing – review & editing.

## References

[B1] Agler M. T. Ruhe J. Kroll S. Morhenn C. Kim S. T. Weigel D.. (2016). Microbial hub taxa link host and abiotic factors to plant microbiome variation. PloS Biol. 14, e1002352. doi: 10.1371/journal.pbio.1002352 26788878 PMC4720289

[B2] Ahmed W. Dai Z. Zhang J. Li S. Ahmed A. Munir S.. (2022). Plant-microbe interaction: mining the impact of native bacillus amyloliquefaciens ws-10 on tobacco bacterial wilt disease and rhizosphere microbial communities. Microbiol. Spectr. 10, e147122. doi: 10.1128/spectrum.01471-22 PMC9430121 35913211

[B3] Albert R. Jeong H. Barabasi A. L. (2000). Error and attack tolerance of complex networks. Nature 406, 378–382. doi: 10.1038/35019019 10935628

[B4] Bachhawat A. K. Thakur A. Kaur J. Zulkifli M. (2013). Glutathione transporters. Biochim. Et Biophys. Acta (Bba) Gen. Subj. 1830, 3154–3164. doi: 10.1016/j.bbagen.2012.11.018 23206830

[B5] Bai Y. Müller D. B. Srinivas G. Garrido-Oter R. Potthoff E. Rott M.. (2015). Functional overlap of the arabidopsis leaf and root microbiota. Nature 528, 364–369. doi: 10.1038/nature16192 26633631

[B6] Brader G. Compant S. Vescio K. Mitter B. Trognitz F. Ma L.. (2017). Ecology and genomic insights into plant-pathogenic and plant-nonpathogenic endophytes. Annu. Rev. Phytopathol. 55, 61–83. doi: 10.1146/annurev-phyto-080516-035641 28489497

[B7] Brandl M. T. (2006). Fitness of human enteric pathogens on plants and implications for food safety. Annu. Rev. Phytopathol. 44, 367–392. doi: 10.1146/annurev.phyto.44.070505.143359 16704355

[B8] Brandl M. T. Mandrell R. E. (2002). Fitness of salmonella enterica serovar thompson in the cilantro phyllosphere. Appl. Environ. Microbiol. 68, 3614–3621. doi: 10.1128/AEM.68.7.3614-3621.2002 12089050 PMC126799

[B9] Carvalho S. D. Castillo J. A. (2018). Influence of light on plant-phyllosphere interaction. Front. Plant Sci. 9. doi: 10.3389/fpls.2018.01482 PMC6194327 30369938

[B10] Chaib De Mares M. Hess J. Floudas D. Lipzen A. Choi C. Kennedy M.. (2015). Horizontal transfer of carbohydrate metabolism genes into ectomycorrhizal amanita. New Phytol. 205, 1552–1564. doi: 10.1111/nph.13140 25407899

[B11] Chase J. M. (2010). Stochastic community assembly causes higher biodiversity in more productive environments. Science 328, 1388–1391. doi: 10.1126/science.1187820 20508088

[B12] Chen M. Li X. Yang Q. Chi X. Pan L. Chen N.. (2014). Dynamic succession of soil bacterial community during continuous cropping of peanut (arachis hypogaea l.). PloS One 9, e101355. doi: 10.1371/journal.pone.0101355 25010658 PMC4092034

[B13] Chi F. Shen S. H. Cheng H. P. Jing Y. X. Yanni Y. G. Dazzo F. B. (2005). Ascending migration of endophytic rhizobia, from roots to leaves, inside rice plants and assessment of benefits to rice growth physiology. Appl. Environ. Microbiol. 71, 7271–7278. doi: 10.1128/AEM.71.11.7271-7278.2005 16269768 PMC1287620

[B14] Christie P. J. Atmakuri K. Krishnamoorthy V. Jakubowski S. Cascales E. (2005). Biogenesis, architecture, and function of bacterial type iv secretion systems. Annu. Rev. Microbiol. 59, 451–485. doi: 10.1146/annurev.micro.58.030603.123630 16153176 PMC3872966

[B15] Dai Y. F. Wu X. M. Wang H. C. Li W. H. Cai L. T. Li J. X.. (2022). Spatio-temporal variation in the phyllospheric microbial biodiversity of alternaria alternata-infected tobacco foliage. Front. Microbiol. 13. doi: 10.3389/fmicb.2022.920109 PMC9370072 35966692

[B16] Da Silva A. Ferro J. A. Reinach F. C. Farah C. S. Furlan L. R. Quaggio R. B.. (2002). Comparison of the genomes of two xanthomonas pathogens with differing host specificities. Nature 417, 459–463. doi: 10.1038/417459a 12024217

[B17] Delmotte N. Knief C. Chaffron S. Innerebner G. Roschitzki B. Schlapbach R.. (2009). Community proteogenomics reveals insights into the physiology of phyllosphere bacteria. Proc. Natl. Acad. Sci. 106, 16428–16433. doi: 10.1073/pnas.0905240106 19805315 PMC2738620

[B18] de Santi Ferrara F. I. Oliveira Z. M. Gonzales H. H. S. Floh E. I. S. Barbosa H. R. (2012). Endophytic and rhizospheric enterobacteria isolated from sugar cane have different potentials for producing plant growth-promoting substances. Plant Soil 353, 409–417. doi: 10.1007/s11104-011-1042-1

[B19] Devescovi G. Bigirimana J. Degrassi G. Cabrio L. LiPuma J. J. Kim J.. (2007). Involvement of a quorum-sensing-regulated lipase secreted by a clinical isolate of burkholderia glumae in severe disease symptoms in rice. Appl. Environ. Microbiol. 73, 4950–4958. doi: 10.1128/AEM.00105-07 17557855 PMC1951028

[B20] Dickel F. Münch D. Amdam G. V. Mappes J. Freitak D. (2018). Increased survival of honeybees in the laboratory after simultaneous exposure to low doses of pesticides and bacteria. PloS One 13, e191256. doi: 10.1371/journal.pone.0191256 PMC5791986 29385177

[B21] Ding C. Zhang W. Wang Y. Ding M. Wang X. Li A.. (2022). Study on the differences of phyllosphere microorganisms between poplar hybrid offspring and their parents. Peerj 10, e12915. doi: 10.7717/peerj.12915 35310169 PMC8932310

[B22] Dini-Andreote F. Stegen J. C. van Elsas J. D. Salles J. F. (2015). Disentangling mechanisms that mediate the balance between stochastic and deterministic processes in microbial succession. Proc. Natl. Acad. Sci. U.S.A. 112, E1326–E1332. doi: 10.1073/pnas.1414261112 25733885 PMC4371938

[B23] Edgar R. C. (2013). Uparse: highly accurate otu sequences from microbial amplicon reads. Nat. Methods 10, 996–998. doi: 10.1038/nmeth.2604 23955772

[B24] Edgar R. C. (2004). MUSCLE: Multiple sequence alignment with high accuracy and high throughput. Nucleic Acids Res. 32, 1792–1797. doi: 10.1093/nar/gkh340 15034147 PMC390337

[B25] Facchinelli F. Weber A. P. (2011). The metabolite transporters of the plastid envelope: an update. Front. Plant Sci. 2. doi: 10.3389/fpls.2011.00050 PMC3355759 22645538

[B26] Feng J. Wang F. Liu G. Greenshields D. Shen W. Kaminskyj S.. (2009). Analysis of a blumeria graminis-secreted lipase reveals the importance of host epicuticular wax components for fungal adhesion and development. Mol. Plant Microbe Interact. 22, 1601–1610. doi: 10.1094/MPMI-22-12-1601 19888825

[B27] Forero-Junco L. M. Alanin K. W. Djurhuus A. M. Kot W. Gobbi A. Hansen L. H. (2022). Bacteriophages roam the wheat phyllosphere. Viruses. doi: 10.3390/v14020244. PMC8876510 35215838

[B28] Friedman J. Alm E. J. (2012). Inferring correlation networks from genomic survey data. PloS Comput. Biol. 8, e1002687. doi: 10.1371/journal.pcbi.1002687 23028285 PMC3447976

[B29] Fu M. Bai Q. Zhang H. Guo Y. Peng Y. Zhang P.. (2022). Transcriptome analysis of the molecular patterns of pear plants infected by two colletotrichum fructicola pathogenic strains causing contrasting sets of leaf symptoms. Front. Plant Sci. 13. doi: 10.3389/fpls.2022.761133 PMC8888856 35251071

[B30] Gu S. Wei Z. Shao Z. Friman V. P. Cao K. Yang T.. (2020). Competition for iron drives phytopathogen control by natural rhizosphere microbiomes. Nat. Microbiol. 5, 1002–1010. doi: 10.1038/s41564-020-0719-8 32393858 PMC7116525

[B31] Guindon S. Dufayard J. F. Lefort V. Anisimova M. Hordijk W. Gascuel O. (2010). New algorithms and methods to estimate maximum-likelihood phylogenies: Assessing the performance of PhyML 3.0. Syst. Biol. 59, 307–321. doi: 10.1093/sysbio/syq010 20525638

[B32] Haefele D. M. Lindow S. E. (1987). Flagellar motility confers epiphytic fitness advantages upon pseudomonas syringae. Appl. Environ. Microbiol. 53, 2528–2533. doi: 10.1128/aem.53.10.2528-2533.1987 16347469 PMC204140

[B33] Hajishengallis G. Lamont R. J. (2016). Dancing with the stars: how choreographed bacterial interactions dictate nososymbiocity and give rise to keystone pathogens, accessory pathogens, and pathobionts. Trends Microbiol. 24, 477–489. doi: 10.1016/j.tim.2016.02.010 26968354 PMC4874887

[B34] Hu Q. Tan L. Gu S. Xiao Y. Xiong X. Zeng W. A.. (2020). Network analysis infers the wilt pathogen invasion associated with non-detrimental bacteria. NPJ Biofilms Microbiomes 6, 8. doi: 10.1038/s41522-020-0117-2 32060424 PMC7021801

[B35] Hulin M. T. Rabiey M. Zeng Z. Vadillo D. A. Bellamy S. Swift P.. (2023). Genomic and functional analysis of phage-mediated horizontal gene transfer in pseudomonas syringae on the plant surface. New Phytol. 237, 959–973. doi: 10.1111/nph.18573 36285389 PMC10107160

[B36] Ikeda S. Okazaki K. Takahashi H. Tsurumaru H. Minamisawa K. (2023). Seasonal shifts in bacterial community structures in the lateral root of sugar beet grown in an andosol field in Japan. Microbes Environ. 38. doi: 10.1264/jsme2.ME22071 PMC10037095 36754423

[B37] Jaffar N. S. Jawan R. Chong K. P. (2023). The potential of lactic acid bacteria in mediating the control of plant diseases and plant growth stimulation in crop production - a mini review. Front. Plant Sci. 13. doi: 10.3389/fpls.2022.1047945. PMC9880282 36714743

[B38] James T. Y. Kauff F. Schoch C. L. Matheny P. B. Hofstetter V. Cox C. J.. (2006). Reconstructing the early evolution of fungi using a six-gene phylogeny. Nature 443, 818–822. doi: 10.1038/nature05110 17051209

[B39] Jiménez-Bremont J. F. Marina M. Guerrero-González M. L. Rossi F. R. Sánchez-Rangel D. Rodríguez-Kessler M.. (2014). Physiological and molecular implications of plant polyamine metabolism during biotic interactions. Front. Plant Sci. 5. doi: 10.3389/fpls.2014.00095 PMC3957736 24672533

[B40] Jin X. Rahman M. K. U. Ma C. Zheng X. Wu F. Zhou X. (2023). Silicon modification improves biochar’s ability to mitigate cadmium toxicity in tomato by enhancing root colonization of plant-beneficial bacteria. Ecotoxicol Environ. Saf. 249, 114407. doi: 10.1016/j.ecoenv.2022.114407 36508786

[B41] Jun W. Barahona M. Yue-Jin T. Hong-Zhong D. (2010). Natural connectivity of complex networks. Chin. Phys. Lett. 27, 78902. doi: 10.1088/0256-307X/27/7/078902

[B42] Kay E. Vogel T. M. Bertolla F. Nalin R. Simonet P. (2002). *In situ* transfer of antibiotic resistance genes from transgenic (transplastomic) tobacco plants to bacteria. Appl. Environ. Microbiol. 68, 3345–3351. doi: 10.1128/AEM.68.7.3345-3351.2002 12089013 PMC126776

[B43] Kembel S. W. O'Connor T. K. Arnold H. K. Hubbell S. P. Wright S. J. Green J. L. (2014). Relationships between phyllosphere bacterial communities and plant functional traits in a neotropical forest. Proc. Natl. Acad. Sci. U.S.A. 111, 13715–13720. doi: 10.1073/pnas.1216057111 25225376 PMC4183302

[B44] Kõljalg U. Larsson K. H. Abarenkov K. Nilsson R. H. Alexander I. J. Eberhardt U.. (2005). Unite: a database providing web-based methods for the molecular identification of ectomycorrhizal fungi. New Phytol. 166, 1063–1068. doi: 10.1111/j.1469-8137.2005.01376.x 15869663

[B45] Kong Y. (2011). Btrim: a fast, lightweight adapter and quality trimming program for next-generation sequencing technologies. Genomics 98, 152–153. doi: 10.1016/j.ygeno.2011.05.009 21651976

[B46] Letunic I. Bork P. (2021). Interactive tree of life (iTOL) v5: An online tool for phylogenetic tree display and annotation. Nucleic Acids Res. 49, W293–W296. doi: 10.1093/nar/gkab301 33885785 PMC8265157

[B47] Li F. Liu W. Gao W. Liu Y. Hu Y. (2022). Design and reliability analysis of a novel redundancy topology architecture. Sensors (Basel) 22. doi: 10.3390/s22072582 PMC9002500 35408197

[B48] Li L. Peng S. Wang Z. Zhang T. Li H. Xiao Y.. (2022). Genome mining reveals abiotic stress resistance genes in plant genomes acquired from microbes *via* hgt. Front. Plant Sci. 13. doi: 10.3389/fpls.2022.1025122. PMC9667741 36407614

[B49] Liu H. Jiang J. An M. Li B. Xie Y. Xu C.. (2022). Bacillus velezensis SYL-3 suppresses alternaria alternata and tobacco mosaic virus infecting nicotiana tabacum by regulating the phyllosphere microbial community. Front. Microbiol. 13. doi: 10.3389/fmicb.2022.840318 PMC9366745 35966697

[B50] Lindow S. E. Brandl M. T. (2003). Microbiology of the phyllosphere. Appl. Environ. Microbiol. 69, 1875–1883. doi: 10.1128/AEM.69.4.1875-1883.2003 12676659 PMC154815

[B51] Liu Z. Beskrovnaya P. Melnyk R. A. Hossain S. S. Khorasani S. O'Sullivan L. R.. (2018). A genome-wide screen identifies genes in rhizosphere-associated pseudomonas required to evade plant defenses. Mbio 9. doi: 10.1128/mBio.00433-18 PMC6222131 30401768

[B52] Liu W. Dong N. Zhang X. H. (2012). Overexpression of mlta in edwardsiella tarda reduces resistance to antibiotics and enhances lethality in zebra fish. J. Appl. Microbiol. 112, 1075–1085. doi: 10.1111/jam.2012.112.issue-6 22443589

[B53] Ma B. Hibbing M. E. Kim H. S. Reedy R. M. Yedidia I. Breuer J.. (2007). Host range and molecular phylogenies of the soft rot enterobacterial genera pectobacterium and dickeya. Phytopathology 97, 1150–1163. doi: 10.1094/PHYTO-97-9-1150 18944180

[B54] Maloney A. P. VanEtten H. D. (1994). A gene from the fungal plant pathogen nectria haematococca that encodes the phytoalexin-detoxifying enzyme pisatin demethylase defines a new cytochrome p450 family. Mol. Gen. Genet. Mgg 243, 506–514. doi: 10.1007/BF00284198 8208242

[B55] Maor R. Shirasu K. (2005). The arms race continues: battle strategies between plants and fungal pathogens. Curr. Opin. Microbiol. 8, 399–404. doi: 10.1016/j.mib.2005.06.008 15996507

[B56] Markowitz V. M. Chen I. A. Palaniappan K. Chu K. Szeto E. Grechkin Y.. (2010). The integrated microbial genomes system: An expanding comparative analysis resource. Nucleic Acids Res. 38, D382–D390. doi: 10.1093/nar/gkp887 19864254 PMC2808961

[B57] Mossey P. Hudacek A. Das A. (2010). Agrobacterium tumefaciens type iv secretion protein virb3 is an inner membrane protein and requires virb4, virb7, and virb8 for stabilization. J. Bacteriol. 192, 2830–2838. doi: 10.1128/JB.01331-09 20348257 PMC2876495

[B58] Müller J. Rout S. Leitsch D. Vaithilingam J. Hehl A. Müller N. (2015). Comparative characterization of two nitroreductases from giardia lamblia as potential activators of nitro compounds. Int. J. Parasitol.: Drugs Drug Resistance 5, 37–43. doi: 10.1016/j.ijpddr.2015.03.001 PMC4813764 27099829

[B59] Nielsen K. M. van Elsas J. D. (2001). Stimulatory effects of compounds present in the rhizosphere on natural transformation of acinetobacter sp. Bd413 in soil. Soil Biol. Biochem. 33, 345–357. doi: 10.1016/S0038-0717(00)00147-4

[B60] Ostolaza H. González-Bullón D. Uribe K. B. Martín C. Amuategi J. Fernandez-Martínez X. (2019). Membrane permeabilization by pore-forming rtx toxins: what kind of lesions do these toxins form? Toxins (Basel) 11. doi: 10.3390/toxins11060354 PMC6628442 31216745

[B61] Pinto-Carbó M. Sieber S. Dessein S. Wicker T. Verstraete B. Gademann K.. (2016). Evidence of horizontal gene transfer between obligate leaf nodule symbionts. Isme J. 10, 2092–2105. doi: 10.1038/ismej.2016.27 26978165 PMC4989318

[B62] Poppenberger B. Berthiller F. Lucyshyn D. Sieberer T. Schuhmacher R. Krska R.. (2003). Detoxification of the fusarium mycotoxin deoxynivalenol by a udp-glucosyltransferase from arabidopsis thaliana. J. Biol. Chem. 278, 47905–47914. doi: 10.1074/jbc.M307552200 12970342

[B63] Qiu H. Cai G. Luo J. Bhattacharya D. Zhang N. (2016). Extensive horizontal gene transfers between plant pathogenic fungi. BMC Biol. 14, 41. doi: 10.1186/s12915-016-0264-3 27215567 PMC4876562

[B64] Redford A. J. Fierer N. (2009). Bacterial succession on the leaf surface: a novel system for studying successional dynamics. Microbial Ecol. 58, 189–198. doi: 10.1007/s00248-009-9495-y 19221834

[B65] Reichenbach T. Kalyani D. Gandini R. Svartström O. Aspeborg H. Divne C. (2018). Structural and biochemical characterization of the cutibacterium acnes exo-β-1,4-mannosidase that targets the n-glycan core of host glycoproteins. PloS One 13, e204703. doi: 10.1371/journal.pone.0204703 PMC6160142 30261037

[B66] Remus-Emsermann M. N. P. Schlechter R. O. (2018). Phyllosphere microbiology: at the interface between microbial individuals and the plant host. New Phytol. 218, 1327–1333. doi: 10.1111/nph.15054 29504646

[B67] Richards T. A. Soanes D. M. Jones M. D. M. Vasieva O. Leonard G. Paszkiewicz K.. (2011). Horizontal gene transfer facilitated the evolution of plant parasitic mechanisms in the oomycetes. Proc. Natl. Acad. Sci. 108, 15258–15263. doi: 10.1073/pnas.1105100108 21878562 PMC3174590

[B68] Richards T. A. Talbot N. J. (2013). Horizontal gene transfer in osmotrophs: playing with public goods. Nat. Rev. Microbiol. 11, 720–727. doi: 10.1038/nrmicro3108 24018383

[B69] Roguet A. Laigle G. S. Therial C. Bressy A. Soulignac F. Catherine A.. (2015). Neutral community model explains the bacterial community assembly in freshwater lakes. FEMS Microbiol. Ecol. 91. doi: 10.1093/femsec/fiv125 26472576

[B70] Salanoubat M. Genin S. Artiguenave F. Gouzy J. Mangenot S. Arlat M.. (2002). Genome sequence of the plant pathogen ralstonia solanacearum. Nature 415, 497–502. doi: 10.1038/415497a 11823852

[B71] Schonknecht G. Weber A. P. Lercher M. J. (2014). Horizontal gene acquisitions by eukaryotes as drivers of adaptive evolution. Bioessays 36, 9–20. doi: 10.1002/bies.201300095 24323918

[B72] Sen M. Shah B. Rakshit S. Singh V. Padmanabhan B. Ponnusamy M.. (2011). Udp-glucose 4, 6-dehydratase activity plays an important role in maintaining cell wall integrity and virulence of candida albicans. PloS Pathog. 7, e1002384. doi: 10.1371/journal.ppat.1002384 22114559 PMC3219719

[B73] Sepúlveda-Jiménez G. Rueda-Benítez P. Porta H. Rocha-Sosa M. (2005). A red beet (beta vulgaris) udp-glucosyltransferase gene induced by wounding, bacterial infiltration and oxidative stress. J. Exp. Bot. 56, 605–611. doi: 10.1093/jxb/eri036 15582929

[B74] Shapiro L. R. Scully E. D. Straub T. J. Park J. Stephenson A. G. Beattie G. A.. (2016). Horizontal gene acquisitions, mobile element proliferation, and genome decay in the host-restricted plant pathogen erwinia tracheiphila. Genome Biol. Evol. 8, 649–664. doi: 10.1093/gbe/evw016 26992913 PMC4824170

[B75] Stegen J. C. Lin X. Fredrickson J. K. Chen X. Kennedy D. W. Murray C. J.. (2013). Quantifying community assembly processes and identifying features that impose them. Isme J. 7, 2069–2079. doi: 10.1038/ismej.2013.93 23739053 PMC3806266

[B76] Talavera G. Castresana J. (2007). Improvement of phylogenies after removing divergent and ambiguously aligned blocks from protein sequence alignments. Syst. Biol. 56, 564–577. doi: 10.1080/10635150701472164 17654362

[B77] Tao J. Yu S. Jin J. Lu P. Yang Z. Xu Y.. (2022). The wilt pathogen induces different variations of root-associated microbiomes of plant. Front. Plant Sci. 13. doi: 10.3389/fpls.2022.1023837. PMC9523445 36186049

[B78] Tauch A. Schneiker S. Selbitschka W. Puhler A. van Overbeek L. S. Smalla K.. (2002). The complete nucleotide sequence and environmental distribution of the cryptic, conjugative, broad-host-range plasmid pipo2 isolated from bacteria of the wheat rhizosphere. Microbiology-Sgm 148, 1637–1653. doi: 10.1099/00221287-148-6-1637 12055285

[B79] Temporini E. D. VanEtten H. D. (2004). An analysis of the phylogenetic distribution of the pea pathogenicity genes of nectria haematococca mpvi supports the hypothesis of their origin by horizontal transfer and uncovers a potentially new pathogen of garden pea: neocosmospora boniensis. Curr. Genet. 46, 29–36. doi: 10.1007/s00294-004-0506-8 15118835

[B80] Truchado P. Gil M. I. Reboleiro P. Rodelas B. Allende A. (2017). Impact of solar radiation exposure on phyllosphere bacterial community of red-pigmented baby leaf lettuce. Food Microbiol. 66, 77–85. doi: 10.1016/j.fm.2017.03.018 28576376

[B81] Vanga B. R. Ramakrishnan P. Butler R. C. Toth I. K. Ronson C. W. Jacobs J. M. E.. (2015). Mobilization of horizontally acquired island 2 is induced in planta in the phytopathogen pectobacterium atrosepticum scri1043 and involves the putative relaxase eca0613 and quorum sensing. Environ. Microbiol. 17, 4730–4744. doi: 10.1111/1462-2920.13024 26271942

[B82] Vass M. Székely A. J. Lindström E. S. Langenheder S. (2020). Using null models to compare bacterial and microeukaryotic metacommunity assembly under shifting environmental conditions. Sci. Rep. 10, 2455. doi: 10.1038/s41598-020-59182-1 32051469 PMC7016149

[B83] Vélëz H. Glassbrook N. J. Daub M. E. (2008). Mannitol biosynthesis is required for plant pathogenicity by alternaria alternata. FEMS Microbiol. Lett. 285, 122–129. doi: 10.1111/fml.2008.285.issue-1 18549402

[B84] Vogel C. Innerebner G. Zingg J. Guder J. Vorholt J. A. (2012). Forward genetic in planta screen for identification of plant-protective traits of sphingomonas sp. Strain fr1 against pseudomonas syringae dc3000. Appl. Environ. Microbiol. 78, 5529–5535. doi: 10.1128/AEM.00639-12 22660707 PMC3406163

[B85] Voigt C. A. Schäfer W. Salomon S. (2005). A secreted lipase of fusarium graminearum is a virulence factor required for infection of cereals. Plant J. For Cell Mol. Biol. 42, 364–375. doi: 10.1111/j.1365-313X.2005.02377.x 15842622

[B86] Vorholt J. A. (2012). Microbial life in the phyllosphere. Nat. Rev. Microbiol. 10, 828–840. doi: 10.1038/nrmicro2910 23154261

[B87] Wang Z. Fu C. Tian J. Wang W. Peng D. Dai X.. (2022). Responses of the bacterial community of tobacco phyllosphere to summer climate and wildfire disease. Front. Plant Sci. 13. doi: 10.3389/fpls.2022.1050967 PMC9811124 36618666

[B88] Wang W. H. Lai T. X. Wu Y. C. Chen Z. T. Tseng K. Y. Lan C. Y. (2022). Associations of rap1 with cell wall integrity, biofilm formation, and virulence in candida albicans. Microbiol. Spectr. 10, e328522. doi: 10.1128/spectrum.03285-22 PMC9769648 36416583

[B89] Wang Z. Song Y. (2022). Toward understanding the genetic bases underlying plant-mediated “cry for help” to the microbiota. Imeta 1, e8. doi: 10.1002/imt2.8 PMC10989820 38867725

[B90] Watts D. J. Strogatz S. H. (1998). Collective dynamics of ‘small-world’ networks. Nature 393, 440–442. doi: 10.1038/30918 9623998

[B91] Whipps J. M. Hand P. Pink D. A. Bending G. D. (2008). Human pathogens and the phyllosphere. Adv. Appl. Microbiol. 64, 183–221. doi: 10.1016/S0065-2164(08)00407-3 18485286

[B92] Xin X. Kvitko B. He S. Y. (2018). Pseudomonas syringae: what it takes to be a pathogen. Nat. Rev. Microbiol. 16, 316–328. doi: 10.1038/nrmicro.2018.17 29479077 PMC5972017

[B93] Xin X. F. Nomura K. Aung K. Velásquez A. C. Yao J. Boutrot F.. (2016). Bacteria establish an aqueous living space in plants crucial for virulence. Nature 539, 524–529. doi: 10.1038/nature20166 27882964 PMC5135018

[B94] Xu P. Stirling E. Xie H. Li W. Lv X. Matsumoto H.. (2023). Continental scale deciphering of microbiome networks untangles the phyllosphere homeostasis in tea plant. J. Adv. Res. 44, 13–22. doi: 10.1016/j.jare.2022.04.002 36725184 PMC9936419

[B95] Xu N. Zhao Q. Zhang Z. Zhang Q. Wang Y. Qin G.. (2022). Phyllosphere microorganisms: sources, drivers, and their interactions with plant hosts. J. Agric. Food Chem. 70, 4860–4870. doi: 10.1021/acs.jafc.2c01113 35435673

[B96] Yin J. Zhang Z. Zhu C. Wang T. Wang R. Ruan L. (2022). Heritability of tomato rhizobacteria resistant to ralstonia solanacearum. Microbiome 10, 227. doi: 10.1186/s40168-022-01413-w 36517876 PMC9753271

[B97] Yuan M. M. Guo X. Wu L. Zhang Y. Xiao N. Ning D.. (2021). Climate warming enhances microbial network complexity and stability. Nat. Climate Change 11, 343–348. doi: 10.1038/s41558-021-00989-9

[B98] Zhou X. Zhang J. Khashi U Rahman M. Gao D. Wei Z. Wu F.. (2023). Interspecific plant interaction *via* root exudates structures the disease suppressiveness of rhizosphere microbiomes. Mol. Plant 16, 849–864. doi: 10.1016/j.molp.2023.03.009 36935607

