# Supplementary material for: Phyllosphere bacterial community dynamics in response to bacterial wildfire disease: succession and interaction patterns
Source: Front Plant Sci. 2024 Mar 12;15:1331443. doi: 10.3389/fpls.2024.1331443 (PMC10963427; doi:10.3389/fpls.2024.1331443)
Supplement: Supplementary file 1 [file DataSheet_1.pdf]

Supplemented Materials

Succession and interaction of phyllosphere bacterial community responding to summer climate and wildfire disease

Deyuan Peng[1], Zhenhua Wang[1], Jinyan Tian[1], Wei Wang[1], Shijie Guo[1], Xi Dai[1], Huaqun Yin[2,3], and Liangzhi Li[2,3]*

[1] Zhangjiajie Tobacco Company of Hunan Province, Zhangjiajie, China
[2]School of Minerals Processing and Bioengineering, Central South University, Changsha, China
[3]Key Laboratory of Biometallurgy of Ministry of Education, Central South University, Changsha, China

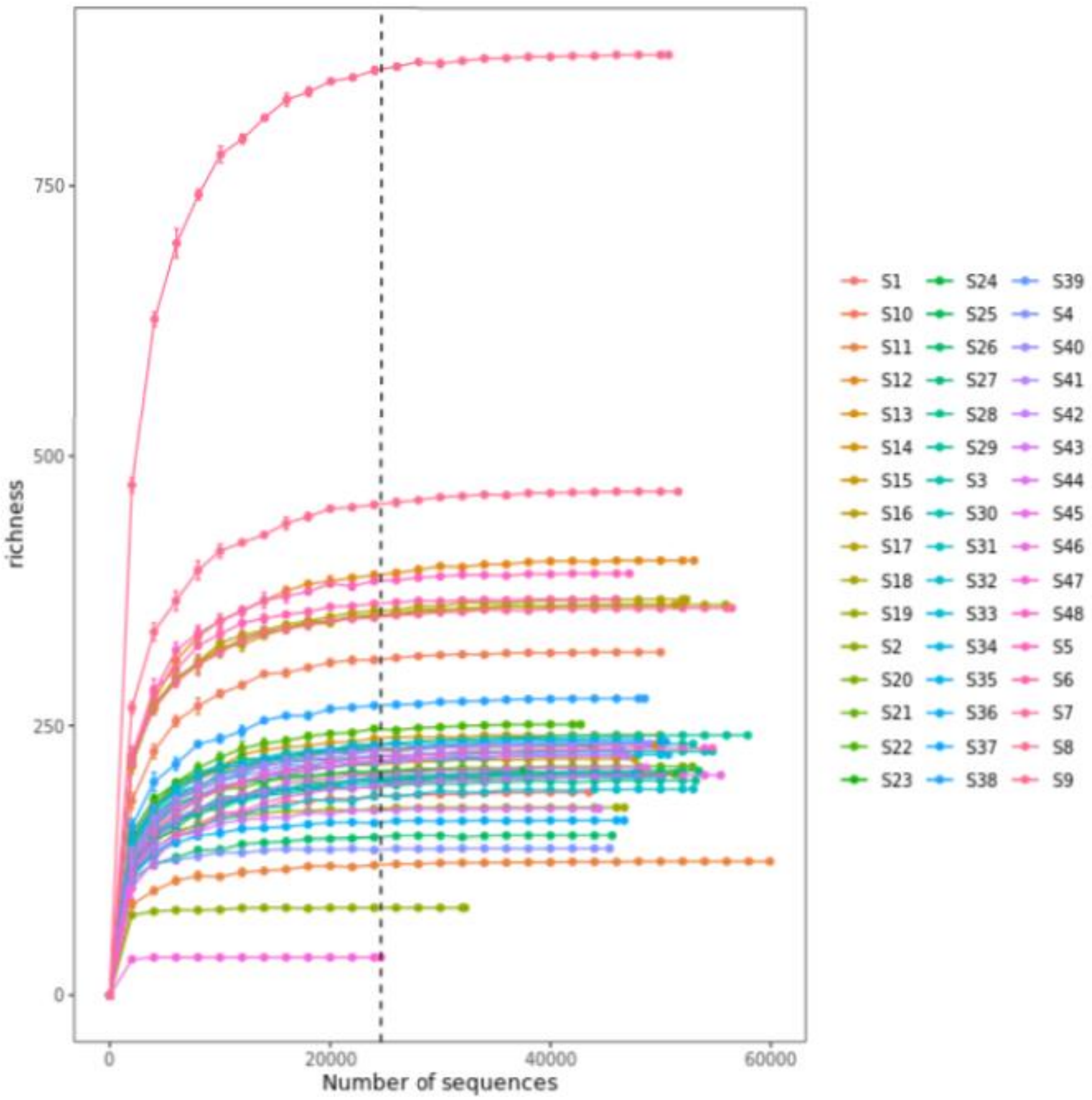

Figure S1: Rarefaction curves to access the richness of bacterial communities.

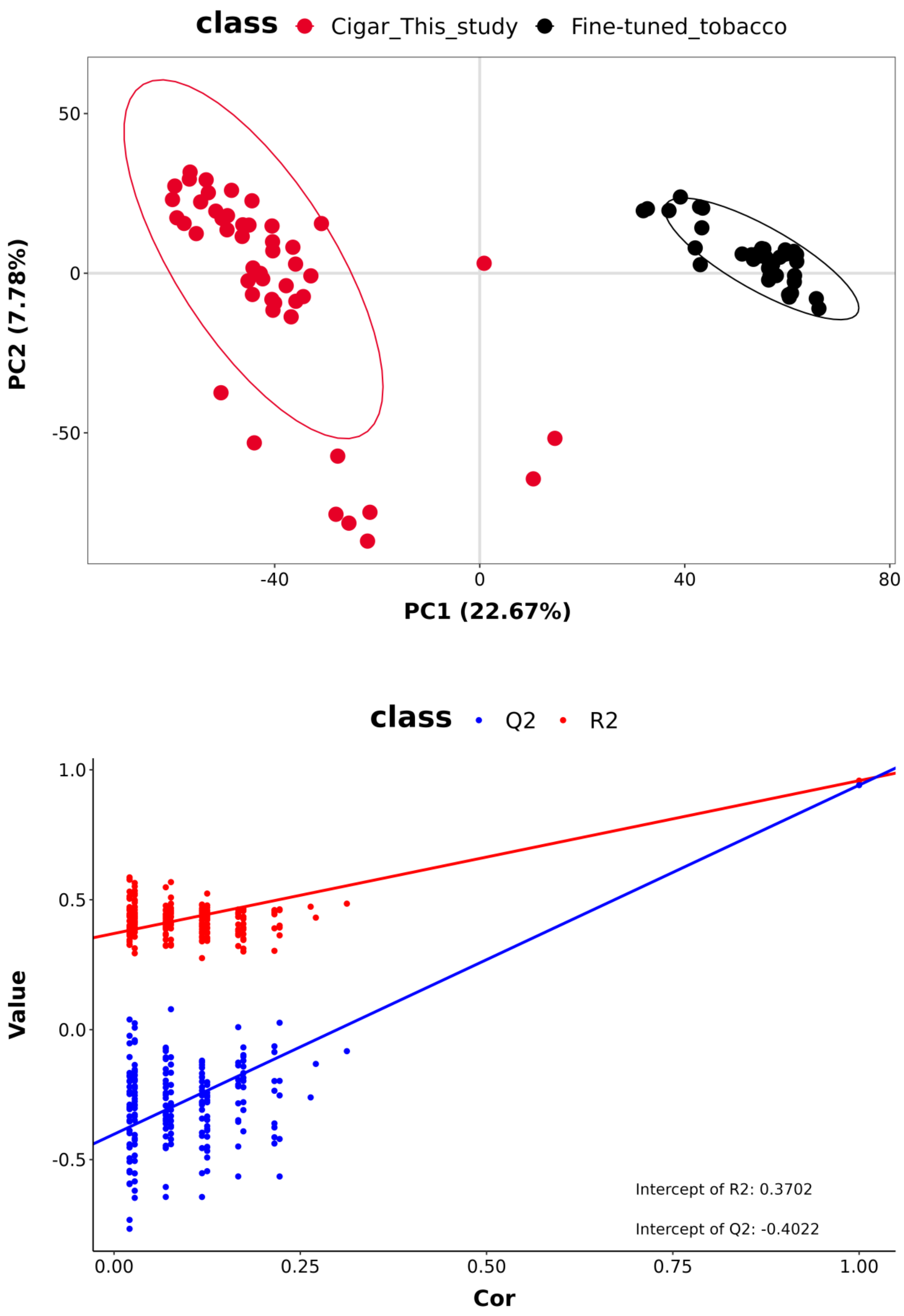

Figure S2: Partial least squares-discriminant analysis (PLS-DA) of the bacterial community structure of cigar tobacco in current study (red) and that of fine-tuned tobacco (black) that we previously reported.

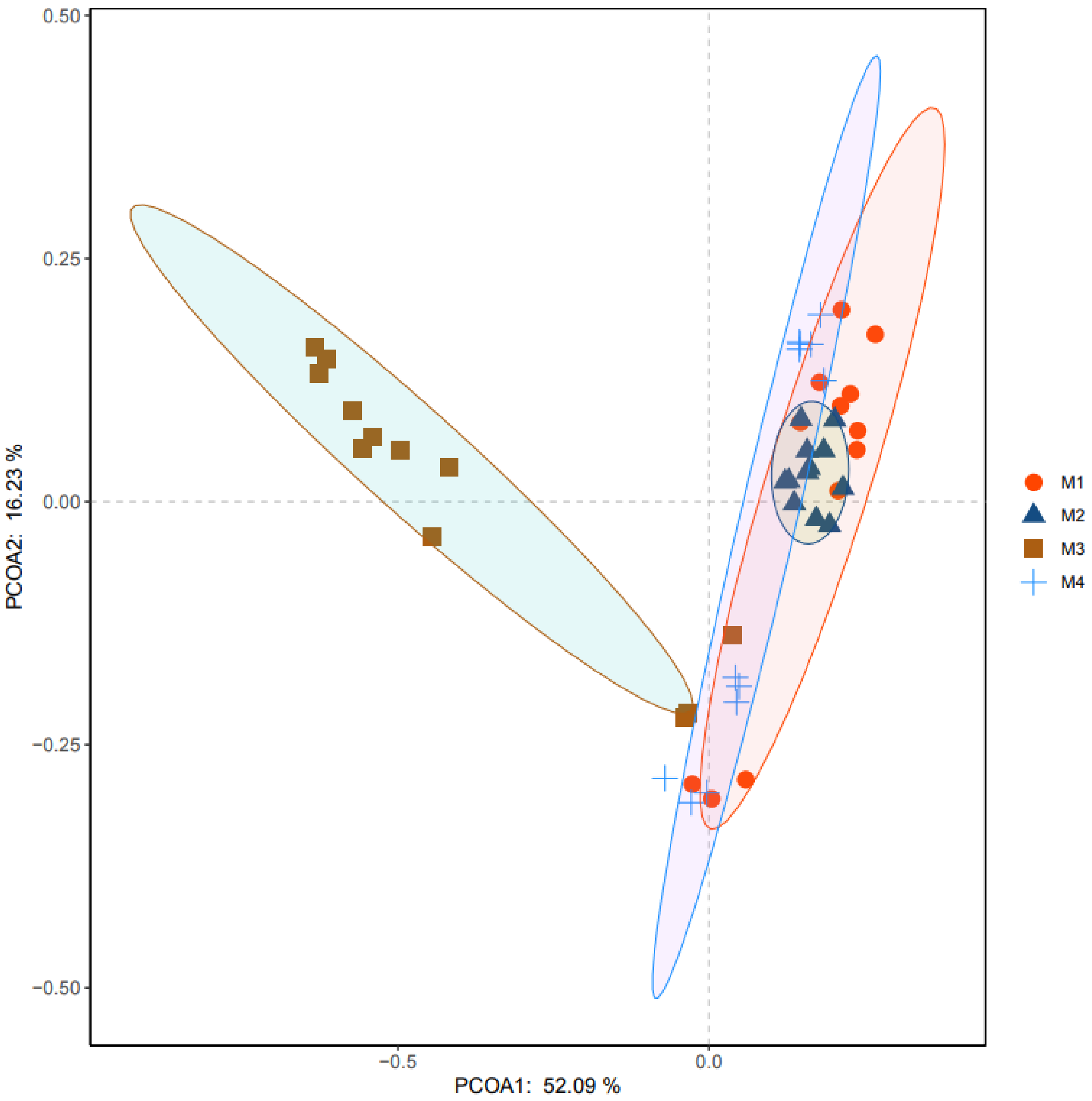

Figure S3: PCoA analysis at the KO level of different time series predicted using PICRUSt2

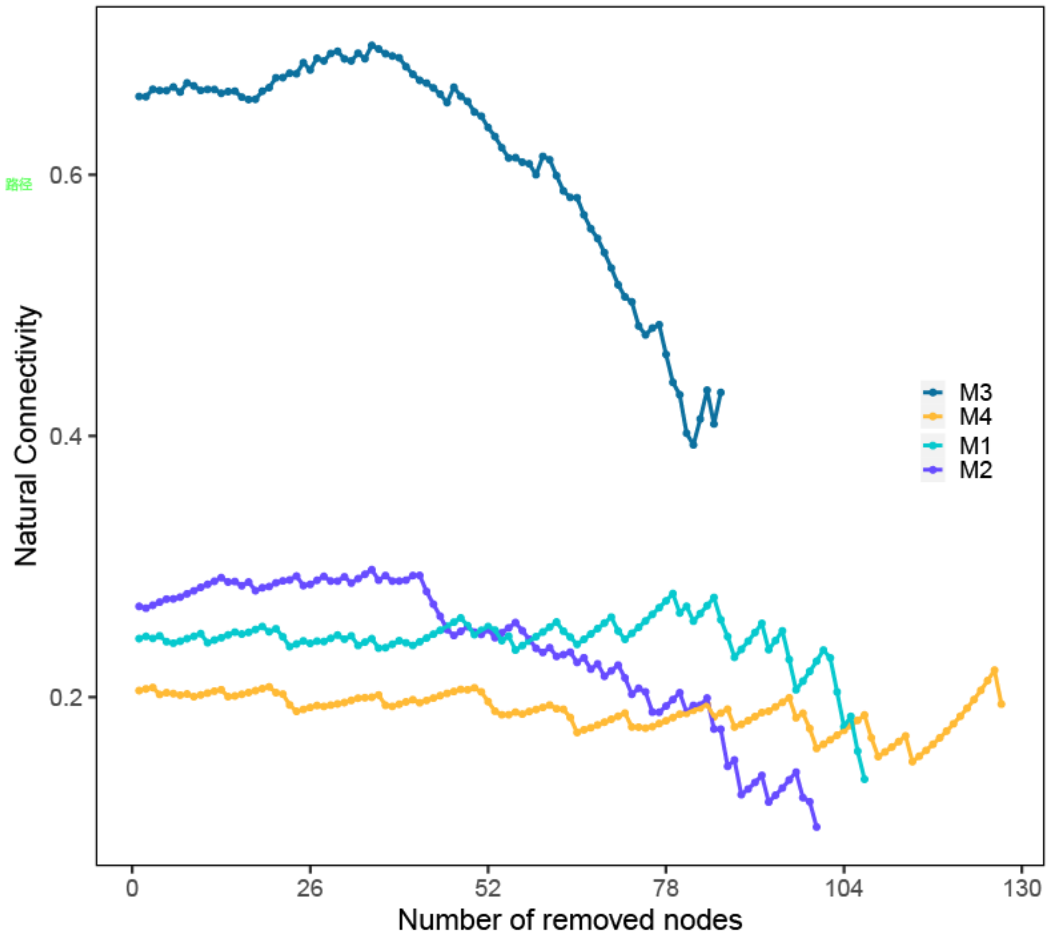

Figure S4: Analysis on the structural robustness of MENs (M1, M2, M3, and M4) by calculating their natural connectivity.
